# Exogenous and endogenous factors affecting stock market transactions: A Hawkes process analysis of the Tokyo Stock Exchange during the COVID-19 pandemic

**DOI:** 10.1371/journal.pone.0301462

**Published:** 2024-04-17

**Authors:** Mariko I. Ito, Yudai Honma, Takaaki Ohnishi, Tsutomu Watanabe, Kazuyuki Aihara

**Affiliations:** 1 Center for Social Complex Systems, Institute of Industrial Science, The University of Tokyo, Meguro-ku, Tokyo, Japan; 2 Graduate School of Artificial Intelligence and Science, Rikkyo University, Toshima-ku, Tokyo, Japan; 3 Graduate School of Economics, The University of Tokyo, Bunkyo-ku, Tokyo, Japan; 4 International Research Center for Neurointelligence, The University of Tokyo Institutes for Advanced Study, The University of Tokyo, Bunkyo-ku, Tokyo, Japan; University of Almeria: Universidad de Almeria, SPAIN

## Abstract

Transactions in financial markets are not evenly spaced but can be concentrated within a short period of time. In this study, we investigated the factors that determine the transaction frequency in financial markets. Specifically, we employed the Hawkes process model to identify exogenous and endogenous forces governing transactions of individual stocks in the Tokyo Stock Exchange during the COVID-19 pandemic. To enhance the accuracy of our analysis, we introduced a novel EM algorithm for the estimation of exogenous and endogenous factors that specifically addresses the interdependence of the values of these factors over time. We detected a substantial change in the transaction frequency in response to policy change announcements. Moreover, there is significant heterogeneity in the transaction frequency among individual stocks. We also found a tendency where stocks with high market capitalization tend to significantly respond to external news, while their excitation relationship between transactions is weak. This suggests the capability of quantifying the market state from the viewpoint of the exogenous and endogenous factors generating transactions for various stocks.

## Introduction

Trade transactions are performed on a micro- to millisecond timescale in financial markets worldwide [1–3]. Studies have been conducted to analyze the transaction frequency [4–10] in addition to the price change per time unit. This has been achieved by taking advantage of the development of high computational power to process precise data for the time at which transactions occurred [10].

Transactions in the stock market do not regularly occur, and their temporal distribution exhibits various patterns reflecting traders’ strategies, sentiments, and news. An example of temporal heterogeneity in transaction frequency is intraday variation, where transactions frequently occur around the start and end of each stock market session [6, 11]. On shorter timescales, we can observe another pattern, that is, temporal clustering of transactions, which can be a result of an instantaneous increase in transaction intensity among investors [7, 10, 12]. On the other hand, such an intensity increase can be caused by an excitation relationship between trading [4, 7]. Investors follow preceding trades strategically or rationally as well as blindly, leading to herding behavior [13, 14]. On the contrary, news and events occurring outside the market, such as disasters, can increase transaction intensity [7, 15]. Additionally, in the present market, high-frequency traders (HFTs) have entered and placed automated orders using computers on a short timescale [1, 3, 16]. Thus, the temporal distribution of transactions exhibits a heterogeneous nature, reflecting the excitation relationship between trading and external information, that is, the endogenous and exogenous factors generating transactions, at various timescales.

The *point process* represents the temporal evolution of transactions. Analyses of point processes have been carried out to infer the strength of the endogenous and exogenous factors generating transactions particularly based on the Hawkes process model. Generally, a point process is defined as a series of time points at which each event occurs. Applications of point process analyses vary, and an example of such an application is financial phenomena [4, 5, 17]. For example, volatility clustering is one focus of these analyses, where the point processes of large returns or price changes are often considered [5, 18, 19]. Particularly, *the Hawkes process* model assumes that events can be generated by the self-excitation effect of past events [4, 5, 20, 21]. By adopting Hawkes process modeling for transaction data, we can estimate values including the *branching ratio* and *background intensity*. The former value represents the strength of self-excitation between transactions (i.e., the endogenous factor determining transaction frequency), and the latter exhibits other factors generating transactions, which can reflect exogenous factors, such as the effect of news on transaction frequency [4, 5, 7].

Recent studies have attempted to capture temporal changes in the strength of endogenous and exogenous factors, whereas conventional studies assume that these are temporally constant over a certain period [6–8, 11]. Particularly, a market temporally experiencing high self-excitation is interpreted as being in an unstable situation at that moment [5, 8].

Our interest is in evaluating the nature of the transaction frequency for various stocks in a market from the viewpoint of temporal changes in the endogenous and exogenous factors that generate transactions. For a point process of transactions, the temporal salience of self-excitation and background intensity can reflect investors’ sentiments to follow preceding transactions and the arrival of big news in stock markets, respectively. The differences in the responses of stocks to these factors can also be considered. Some stocks may frequently exhibit herding behavior in transactions, whereas others may significantly reflect the news. The previous study also suggested the variation of stocks in the extent to which they are involved in financial instability [22]. This study aims to outline the market state by comparing the time-varying endogenous and exogenous factors generating transactions among various stocks.

In this study, we attempted to outline the state of a market by considering examples of trade transactions on the Tokyo Stock Exchange (TSE) over several days during the financial turmoil in March 2020. In that month, stock markets were disrupted worldwide by the COVID-19 pandemic and experienced clashes in the middle of the month [23, 24]. To control the outbreak, governments locked down the country, banned travel, and adopted social distancing and quarantine policies [25]. Previous studies have suggested that such governmental measures provoke investor anxiety because they can suppress not only the spread of infectious diseases but also socioeconomic activities [26–31]. Therefore, March 2020 is considered a period when the world stock markets experienced uncertainty, greatly affecting investors’ sentiments and transactions and the frequent arrival of news, each of which is associated with the aforementioned endogenous and exogenous factors for generating transactions. To the best of our knowledge, no study has evaluated these factors for various stocks in markets during the March 2020 turmoil using the Hawkes process model. This study addresses two research questions taking as an example the transaction frequency in the March 2020 turmoil: (1) how the estimated endogenous and exogenous factors for various stocks outline the investors’ response against uncertainty and news; (2) whether we can characterize stocks based on each of these factors in terms of the transaction frequency.

We modified the method of Wehrli and Sornette (2022) [8] to estimate the temporal changes in endogenous and exogenous factors with the transaction frequency. They proposed an EM algorithm that considered the *branching structure* [8], which represents the lineage relationship among events, namely, the “which event generated which” relationship [8, 32]. By referring to this method, we developed an algorithm to calculate the dynamic changes in the self-excitation strength and background intensity that are consistent with the hidden branching structure between events, causing a more feasible estimation. However, to outline the transaction frequency in the market, we further modified the previously proposed method as follows. First, we reduced the number of parameters to be estimated by assuming a simple Hawkes model and relatively rough temporal changes in the strength of endogenous and exogenous factors. This modification enabled us to reduce the computational cost of analyzing more than a thousand point processes for various stocks throughout the month. Second, we considered the temporal continuity of the strength of self-excitation and the background intensity more carefully than the previous study and simultaneously estimated these parameters at various time points by solving optimization problems with numerous variables. These modifications allow us to outline the nature of a market by obtaining a temporally rougher but more feasible estimation than the original method.

The main contributions of this study are as follows. First, we introduced a novel EM algorithm for the estimation of exogenous and endogenous factors that specifically addresses the interdependence of the values of these factors over time. Our algorithm meticulously accounts for the temporal continuity of these factors, setting it apart from seminal works, such as Wehrli & Sornette (2022), which treat the values of these factors at each time point as independent of each other before curve fitting. Our mathematical model aligns more closely with the realities of time-series events and offers a robust numerical method for parameter estimation by integrating temporal interdependence. Second, applying this algorithm to a broad dataset covering all weekdays in March 2020 for 27 stocks allowed us to analyze over a thousand point processes, providing a detailed overview of the transactions in the TSE during the initial stage of the COVID-19 pandemic. Third, this study unveils unique insights into market dynamics, especially uncovering consistent patterns related to the firm’s size in the influence of exogenous news and endogenous transaction behaviors. Our study delves into how these factors influence investor reactions to news announcements and the enhanced excitation relationship between transactions following measures against financial instability. By applying our comprehensive methodology to such an expansive dataset for the first time, we offer a new perspective on deciphering market reactions to various exogenous and endogenous factors.

In the remainder of this paper, we introduce the Hawkes model and the estimation method, including the EM algorithm in the Methods section. The results of our analyses on the transaction frequency for various stocks are presented in the Results section and discussed in the Discussion section.

## Methods

### Hawkes model

We analyzed the temporal heterogeneity in the transaction frequency based on the idea of point processes. Point process analyses consider the sequence {*t*_*i*_} of timestamps at which each event occurs. *t*_*i*_ denotes the time at which the *i*th event occurred in the observation period [0, *T*] (*t*_*i*_ ∈ [0, *T*]). The Hawkes process, which is a self-excitation process, assumes that an event can trigger subsequent events, eventually generating event clusters.

In the Hawkes process, the event occurrence rate λ(*t*) at time *t*, called the intensity function, is often expressed as

$$\begin{array}{c} \lambda \left(t\right)=\mu +\sum_{i:t_{i}<t}\eta h\left(t-t_{i}\right). \end{array}$$ (1)

The contribution of endogenous factors to generating events is represented by the second term on the right side of Eq (1), and the intensity at time *t* is increased by the accumulation of the effects of preceding events occurring at {*t*_*i*_;*t*_*i*_ < *t*}. The effect of each event on the increasing future intensity is assumed to decline with time, where the memory kernel *h*(*t*) represents such an attenuation of the excitation effect. In this study, we assumed *h*(*t*) = *b* exp(−*bt*) with *b* > 0. The parameter *η*, called the branching ratio, can be regarded as the expected number of events that an event can generate in the future. The first term *μ* in Eq (1) is called the background intensity and represents the baseline intensity. Factors that generate events other than the preceding ones, called exogenous factors, can be incorporated into *μ*. If we regard an event as a transaction in the market, *μ* can represent the effect of external shocks on transactions, such as news, accidents, volatility of indices, or daily trends in their frequency [7].

In this study, we considered temporal changes in the background intensity and branching ratio, assuming the following intensity function for transaction frequency [8]:

$$\begin{array}{c} \lambda \left(t\right)=\mu \left(t\right)+\sum_{i:t_{i}<t}\eta \left(t_{i}\right)h\left(t-t_{i}\right)\;\text{with}\;h\left(t\right)=b\;\text{exp}\left(-bt\right). \end{array}$$ (2)

### EM algorithm considering branching structure

To estimate the temporal changes in the background intensity *μ*(*t*) and branching ratio *η*(*t*) in Eq (2) of the Hawkes process, we referred to the EM algorithm that considers the *branching structure* provided by Wehrli and Sornette (2022) [8]. In this section, we present the concept of the EM algorithm. The detailed calculation associated with this algorithm (e.g., the derivation of the log-likelihood function) is shown in Section S.1 in S1 Text.

The branching structure denotes the lineage relationship between all events. This structure can be represented by matrix *Z* as follows:

$$\begin{array}{c} Z_{i,j}=\{\begin{array}{c} 1,\;\text{if}\;i\neq j\;\text{and}\;\text{event}\;x_{i}\;\text{is}\;\text{generated}\;\text{by}\;\text{event}\;x_{j}\text{,}\\ 1,\;\text{if}\;i=j\;\text{and}\;\text{event}\;x_{i}\;\text{is}\;\text{exogenously}\;\text{generated,}\\ 0,\;\text{otherwise.} \end{array} \end{array}$$ (3)

*x*_*i*_ is an event that occurs at time *t*_*i*_. We further consider the probabilities *π*_*i*,*j*_ (*i* ≠ *j*) and *π*_*i*,*i*_ of event *x*_*i*_ being endogeneously generated by another event *x*_*j*_ and exogeneously generated at time *t*_*i*_, respectively (∑_*j*<*i*_ *p*_*i*,*j*_ + *p*_*i*,*i*_ = 1). The Hawkes process, defined by the intensity function of Eq (1) is equivalent to a branching process where an event is exogenously generated with rate *μ*, and each event further generates another one, or reproduces an offspring, with the rate *ηh*(*t* − *t*_*i*_) at time *t* [32]. Considering this relationship between the Hawkes and branching processes, *π* can be calculated as

$$\begin{array}{c} \pi_{i,i}=\frac{\mu_{i}}{\lambda (t_{i})}, \end{array}$$ (4)

$$\begin{array}{c} \pi_{i,j}=\frac{\eta_{j}h\left(t_{i}-t_{j}\right)}{\lambda (t_{i})}, \end{array}$$ (5)

where *μ*_*i*_ and *η*_*i*_ represent background intensity *μ*(*t*_*i*_) and branching ratio *η*(*t*_*i*_) at which the *i*th event occurs, respectively.

In the EM algorithm, the estimated parameters *θ* are ({*μ*_*i*_}, {*η*_*i*_}, *b*), which are recursively updated until they become consistent before and after an update. Specifically, in the *l*th update, we derive $\theta^{\left(l\right)}=(\left\{\mu_{i}^{\left(l\right)}\right\},\left\{\eta_{i}^{\left(l\right)}\right\},b^{\left(l\right)})$ based on *θ*^(*l*−1)^ (*l* = 1, 2, …), where *θ*^(*l*)^ is the estimated values of the parameters at the *l*th update of the EM algorithm. Each update consisted of E- and M-steps.

In the E-step, the expectation of the log-likelihood of *θ* over the possible branching structures is calculated. The probability of observing a branching structure *Z* depends on *π*_*i*,*j*_, which is derived using {*t*_*i*_} and *θ* (Eqs (4) and (5)). By utilizing the EM algorithm, we calculated *π*_*i*,*j*_ based on the last estimation *θ*^(*l*−1)^ of the parameters and then derived the expected log-likelihood of *θ*, ${\text{E}}_{Z|\{t_{i}\},\theta^{\left(l-1\right)}}$ [log *L*(*θ*|{*t*_*i*_}, *Z*)](≕ *Q*(*θ*|{*t*_*i*_}, *θ*^(*l*−1)^)), in the *l*th step of the algorithm. *Q*(*θ*|{*t*_*i*_}, *θ*^(*l*−1)^) is calculated as follows:

$$\begin{array}{c} Q(\theta |\left\{t_{i}\right\},\theta^{\left(l-1\right)})\\ =n_{T}\;\text{log}\;\Delta +\sum_{i=1}^{n_{T}}\{\pi_{i,i}{|}_{\theta^{\left(l-1\right)}}\;\text{log}\;\mu_{i}+\sum_{j;t_{j}<t_{i}}(\pi_{i,j}{|}_{\theta^{\left(l-1\right)}}\;\text{log}\;(\eta_{j}h\left(t_{i}-t_{j}\right)))\}-\int_{0}^{T}\lambda \left(t\right)dt, \end{array}$$ (6)

where *n*_*T*_ is the number of events during the observation period. The first term in Eq (6) is constant.

In the M-step, *θ*^(*l*)^ is given by *θ* that maximizes the expected log-likelihood *Q*(*θ*|*w*, *θ*^(*l*−1)^) calculated in the E-step as follows:

$$\begin{array}{c} \theta^{\left(l\right)}=\underset{\theta }{\text{arg}\;\text{max}}[Q(\theta |\left\{t_{i}\right\},\theta^{\left(l-1\right)})]. \end{array}$$ (7)

In the M-step, we did not adopt the procedure provided by Wehrli and Sornette (2022) but solved the maximization problem by developing another method that more precisely considers the continuity of *η*(*t*). The previous study obtained a candidate for $\eta_{i}^{\left(l\right)}$ in the *M*-step by analytically solving ∂*Q*(*θ*|{*t*_*i*_}, *θ*^(*l*)^)/∂*η*_*i*_ = 0, where *η*_*i*_ is assumed to be independent of *η*_*j*_ for *i* ≠ *j*. With this assumption of independent variables, $\left\{\left(t_{i},\eta_{i}^{\left(l\right)}\right)\right\}$ is regarded as noisy. A spline function *f*(*t*) is fitted to $\left\{\left(t_{i},\eta_{i}^{\left(l\right)}\right)\right\}$ after statistical consideration to eliminate unnecessary points of $\left\{\left(t_{i},\eta_{i}^{\left(l\right)}\right)\right\}$ for a feasible estimation of *η*(*t*), avoiding overfitting [33]. Finally, $\eta_{i}^{\left(l\right)}$ is replaced with *f*(*t*_*i*_). Although the M-step of the previous study can reduce the computational cost of estimating the function *η*(*t*) by analytically deriving a candidate of $\left\{\eta_{i}^{\left(l\right)}\right\}$, the following concerns are yet to be addressed. The branching ratio *η*(*t*) represents the extent to which an event at *t* induces a future event. Such an extent reflects the group’s minds of investors and should change continuously. Considering the continuity of *η*(*t*), the maximization of the log-likelihood with respect only to *η*_*i*_ can cause its misestimation, even if we fit a continuous curve, as in the previous study. Furthermore, the estimation of the transition of the branching ratio and background intensity can become more feasible by considering the dependency among all parameters ({*η*_*i*_}, {*μ*_*i*_}, *b*).

**Fig 1:**
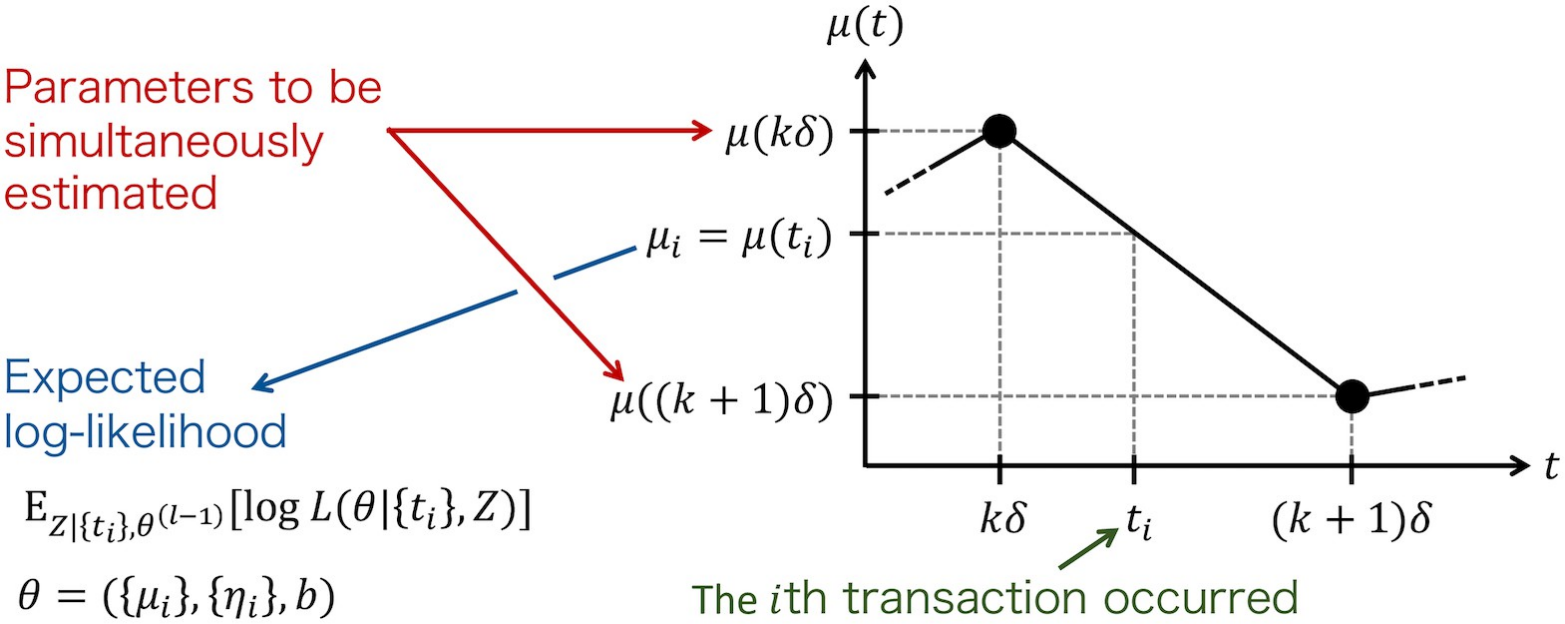
Estimation of *μ*(*t*) and *η*(*t*). We estimated *μ*(*kδ*) for *k* = 0, 1, …, *K* simultaneously, where the background intensity when events occurred, *μ*_*i*_ (= *μ*(*t*_*i*_)), is provided by the linear interpolation of the data {*μ*(*kδ*)}. The branching ratio *η*(*kδ*) (*k* = 0, 1, …, *K*) and *η*(*t*_*i*_) are also obtained in the same manner.

We attempted to maximize *Q*(*θ*|{*t*_*i*_}, *θ*^(*l*)^) with respect to the values of *η*(*t*) and *μ*(*t*) at multiple time points and *b* simultaneously. For the maximization, we used LocalSolver software (https://www.localsolver.com/), which is suitable to optimization problems with numerous variables. Specifically, we obtained *μ*(*kδ*) and *η*(*kδ*) (*k* = 0, 1, …, *K*), where the observation period is separated into *K* subintervals of width *δ* = *T*/*K* (Fig 1).

$$\begin{array}{c} \left(\{\mu (k\delta )\},\{\eta (k\delta )\},b\right)\\ =\underset{\left(\{\mu (k\delta )\},\{\eta (k\delta )\},b\right)}{\text{arg}\;\text{max}}[\sum_{i=0}^{n_{T}-1}\{\pi_{i,i}\;\text{log}\;\mu_{i}+\sum_{j=0}^{i-1}(\pi_{i,j}(\text{log}\;\eta_{j}+\text{log}\;b-b\left(t_{i}-t_{j}\right)))\}\\ \;-\int_{0}^{T}\mu \left(t\right)dt-\sum_{i=0}^{n_{T}-1}\eta_{i}\{1-\text{exp}(-b(T-t_{i}))\}], \end{array}$$ (8)

where the background intensity and branching ratio when events occur, *μ*_*i*_ and *η*_*i*_, are provided through the linear interpolation of the data {*μ*(*kδ*)} and {*η*(*kδ*)}, respectively. Namely,

$$\begin{array}{c} \mu_{i}=\frac{\left(t_{i}-k\delta \right)\mu ((k+1)\delta )+(\left(k+1\right)\delta -t_{i})\mu (k\delta )}{\delta }, \end{array}$$ (9)

$$\begin{array}{c} \eta_{i}=\frac{\left(t_{i}-k\delta \right)\eta ((k+1)\delta )+(\left(k+1\right)\delta -t_{i})\eta (k\delta )}{\delta }, \end{array}$$ (10)

where *k* = ⌊*t*_*i*_/*δ*⌋ and ⌊*⌋ are the floor function. The algorithm to estimate *μ*(*t*) and *η*(*t*) is summarized in Algorithm 1.

**Algorithm 1** EM algorithm.

1: Initialization of *θ*^(0)^ = ({*μ*^(0)^(*kδ*)}, {*η*^(0)^(*kδ*)}, *b*^(0)^)

2: $\mu_{i}^{\left(0\right)}=r_{k}\mu^{\left(0\right)}((k+1)\delta )+\left(1-r_{k}\right)\mu^{\left(0\right)}(k\delta )$, *r*_*k*_ = (*t*_*i*_ − *kδ*)/*δ*, *k* = ⌊*t*_*i*_/*δ*⌋, *i* = 1, …, *n*_*T*_

3: $\eta_{i}^{\left(0\right)}=r_{k}\eta^{\left(0\right)}((k+1)\delta )+\left(1-r_{k}\right)\eta^{\left(0\right)}(k\delta )$, *r*_*k*_ = (*t*_*i*_ − *kδ*)/*δ*, *k* = ⌊*t*_*i*_/*δ*⌋, *i* = 1, …, *n*_*T*_

4: *l* = 1

5: **while** |*θ*^(*l*)^ − *θ*^(*l*−1)^| > *ϵ* or *l* = 1 **do**

6:  $\lambda_{i}=\mu_{i}^{\left(l-1\right)}+\sum_{j;t_{j}<t_{i}}\eta_{j}^{\left(l-1\right)}b^{\left(l-1\right)}\text{exp}(-b^{\left(l-1\right)}\left(t_{i}-t_{j}\right))$, *i* = 1, …, *n*_*T*_

7:  $\pi_{i,i}=\mu_{i}^{\left(l-1\right)}/\lambda_{i}$, $\pi_{i,j}=\{\eta_{j}^{\left(l-1\right)}b^{\left(l-1\right)}\text{exp}(-b^{\left(l-1\right)}\left(t_{i}-t_{j}\right))\}/\lambda_{i}$, *i* = 1, …, *n*_*T*_, *j* = 1, …, *i* − 1

8:  ({*μ*^(*l*)^(*kδ*)}, {*η*^(*l*)^(*kδ*)}, *b*^(*l*)^)

9:   $=\underset{\left(\{\mu (k\delta )\},\{\eta (k\delta )\},b\right)}{\text{arg}\;\text{max}}[\sum_{i=1}^{n_{T}}\{\pi_{i,i}\;\text{log}\;\mu_{i}+\sum_{j=1}^{i-1}(\pi_{i,j}(\text{log}\;\eta_{j}+\text{log}\;b-b\left(t_{i}-t_{j}\right)))\}$

10:   $\;-\int_{0}^{T}\mu \left(t\right)dt-\sum_{i=1}^{n_{T}}\eta_{i}\{1-\text{exp}(-b(T-t_{i}))\}]$,

11:  where

12:   *μ*_*i*_ = *r*_*k*_*μ*((*k* + 1)*δ*) + (1 − *r*_*k*_)*μ*(*kδ*), *r*_*k*_ = (*t*_*i*_ − *kδ*)/*δ*, *k* = ⌊*t*_*i*_/*δ*⌋, *i* = 1, …, *n*_*T*_,

13:   *η*_*i*_ = *r*_*k*_*η*((*k* + 1)*δ*) + (1 − *r*_*k*_)*η*(*kδ*), *r*_*k*_ = (*t*_*i*_ − *kδ*)/*δ*, *k* = ⌊*t*_*i*_/*δ*⌋, *i* = 1, …, *n*_*T*_,

14: $\mu_{i}^{\left(l\right)}=r_{k}\mu^{\left(l\right)}((k+1)\delta )+\left(1-r_{k}\right)\mu^{\left(l\right)}(k\delta )$, *r*_*k*_ = (*t*_*i*_ − *kδ*)/*δ*, *k* = ⌊*t*_*i*_/*δ*⌋, *i* = 1, …, *n*_*T*_

15: $\eta_{i}^{\left(l\right)}=r_{k}\eta^{\left(l\right)}((k+1)\delta )+\left(1-r_{k}\right)\eta^{\left(l\right)}(k\delta )$, *r*_*k*_ = (*t*_*i*_ − *kδ*)/*δ*, *k* = ⌊*t*_*i*_/*δ*⌋, *i* = 1, …, *n*_*T*_

16: *l* = *l* + 1

17: **end while**

### Data and empirical analysis

We analyzed stock market transaction data of the TSE. The analyzed data were JPX Data Cloud (http://db-ec.jpx.co.jp/?__lang=en) Cash Market Data (Tick) for March 2020. The data included records of executions for 21 days on TSE. Each transaction data point shows the stock code, industry code, time, price, and volume, among others. Time was recorded with the precision of 10^−6^ s. We also utilized the data of End-of-Month Market capitalization by Individual Issue (Domestic Equities) (https://db-ec.jpx.co.jp/category/C024/) to evaluate the characteristics of stocks.

**Fig 2:**
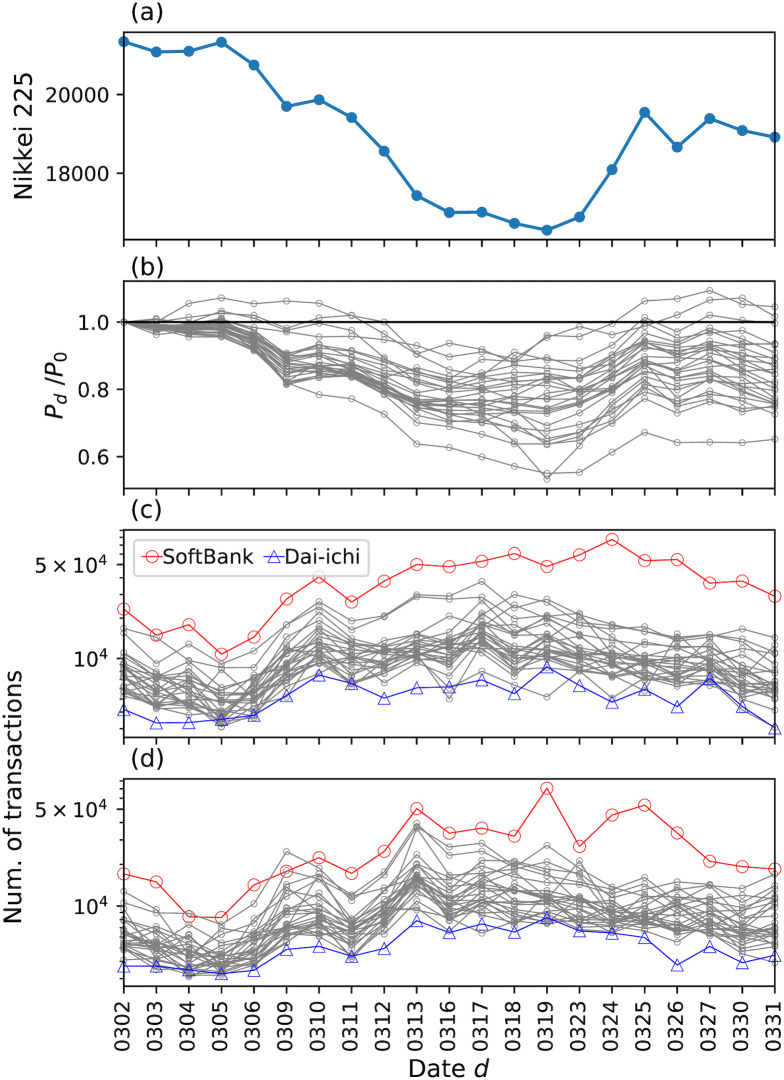
Price change and the number of transactions. (a) and (b) show the price change in March 2020. The horizontal axis exhibits the date. In panel (a), the closing price of the Nikkei Stock Average [34] is shown. In panel (b), for each analyzed stock, the normalized price *P*_*d*_/*P*_0_, where *P*_*d*_ and *P*_0_ are the closing prices on date *d* and March 2, respectively, is shown on the vertical axis. The numbers of transactions in the morning and afternoon sessions are shown in (c) and (d), respectively. The horizontal and vertical axes show the date and the number of transactions, respectively, on that date. The vertical axis is logarithmically scaled. Each plot corresponds to a stock. The number of transactions for SoftBank (Dai-ichi), which has the largest (smallest) number of transactions in the analyzed period, is highlighted in red circles (blue triangles).

In our analysis, we focused on stocks with sufficient multiple transactions. We selected 27 stocks (Table 1) from the first section of TSE, each of which had more than 3,000 transactions in both morning and afternoon trading sessions throughout March. The prices of the Nikkei Stock Average and these stocks rapidly declined from the beginning to the middle of March (Fig 2(a) and 2(b)). Fig 2(c) and 2(d) present the number of transactions related to these stocks in March. For almost all analyzed stocks, the number of transactions increased during the period when the prices rapidly declined. Global markets experienced financial turmoil in mid-March due to the COVID-19 pandemic, as mentioned in the Introduction section. According to the “Holistic Review of the March Market Turmoil” reported by the Financial Stability Board (FSB) [23], March 2020 was divided into three periods: the period from February 21 to March 11 when investors’ sentiment shifted to risk-off (“flight to safety”), period from March 11 to 23 when investors sold most assets to increase cash balances (“dash for cash”), and period after March 23 when the market was recovering corresponding to interventions by governments and authorities (“easing of market stress”).

**Table 1:** Stocks and their abbreviations.

Stock name	Abbreviation
SoftBank Group Corp.	SoftBank
Recruit Holdings Co., Ltd.	Recruit
SUMCO Corporation	SUMCO
INPEX Corporation	INPEX
Nomura Holdings, Inc.	Nomura
Marubeni Corporation	Marubeni
Mitsui Fudosan Co., Ltd.	Mitsui RE
KOMATSU Ltd.	KOMATSU
Dai-ichi Life Holdings, Inc.	Dai-ichi
TORAY Industries, Inc.	TORAY
Panasonic Holdings Corporation	Panasonic
Mizuho Financial Group, Inc.	Mizuho
Resona Holdings, Inc.	Resona
Takeda Pharmaceutical Company Limited	Takeda
Mitsubishi UFJ Financial Group, Inc.	MitsubishiUFJ
Shiseido Company, Limited	Shiseido
KDDI Corporation	KDDI
NISSAN Motor Co., Ltd.	NISSAN
HONDA Motor Co., Ltd.	HONDA
CANON Inc.	CANON
ENEOS Holdings, Inc.	ENEOS
Sumitomo Mitsui Financial Group, Inc.	SumitomoMitsui
TOYOTA Motor Corporation	TOYOTA
Murata Manufacturing Co., Ltd.	Murata
SONY Group Corporation	SONY
Mitsubishi Chemical Group Corporation	MitsubishiChem
Nippon Telegraph and Telephone Corporation	NTT

To empirically analyze the occurrence of transactions, we constructed a point process of transactions for each date, trading session, and stock. We considered a transaction as an event and constructed a point process $\left\{t_{i}^{d,s,\text{am}}\right\}$ ($\left\{t_{i}^{d,s,\text{pm}}\right\}$), where $t_{i}^{d,s,\text{am}}$ ($t_{i}^{d,s,\text{pm}}$) refers to the *i*th transaction for stock *s* in the morning (afternoon) trading session. Hereafter, the unit of time *t* is “second”, and time point 0 denotes the starting time of the morning (afternoon) session at 9:00 (12:30). Because both the morning and afternoon sessions have a duration of 2.5 h, the transaction point process is in the time interval of [0, *T*], where *T* = 9, 000.

In addition, for the point process for each stock in each trading session, we eliminated the timestamps at the start and end of each session. This was because the numbers of these timestamps were significantly larger than those of the others. Additionally, external factors outside the observation period, such as news reported during the night, should affect transactions at the start of each session. The characteristics of the transactions at these boundaries may differ from those during the observation period. Therefore, we limited the objective of our analyses to the latter transactions with time stumps other than the boundaries. Even after such preprocessing, the first timestamp was 12 min past the starting point at the latest for each stock.

From each of these point processes, we estimated the temporal changes *μ*(*t*) and *η*(*t*) by applying the EM algorithm described in the previous section. Specifically, we obtained *μ*(*kδ*) and *η*(*kδ*) (*k* = 0, 1, …, *K*) using the EM algorithm, where the observation period [0, *T*] is separated into *K* subintervals of width *δ* = *T*/*K*. We set *K* = 10 (i.e., *δ* has 15 minutes width). Although our assumptions on temporal changes in *μ*(*t*) and *η*(*t*) are rough compared to those in previous studies [6–8], we can reduce the number of parameters to estimate and easily compare *μ*(*t*) or *η*(*t*) between numerous stocks. Therefore, these assumptions are suitable for evaluating *μ*(*t*) and *η*(*t*) for more than a thousand point processes for various stocks to outline transactions in the market.

Regarding the update of parameters $\theta^{\left(l\right)}=(\left\{\mu_{i}^{\left(l\right)}\right\},\left\{\eta_{i}^{\left(l\right)}\right\},b^{\left(l\right)})$ in the EM algorithm, we determined the initial values of *θ*^(0)^ in the EM algorithm by numerically maximizing the log-likelihood function log *L*(*θ*|{*t*_*i*_}) of parameters *θ* given the analyzed point process {*t*_*i*_}. Therefore, our procedure first obtains candidates for *θ* and then securely determines *θ*, which is consistent with a hidden branching structure. We ran LocalSolver for 900 s to numerically maximize the log-likelihood to determine the initial values of the parameters. In the EM algorithm, for each iteration, the numerical maximization of the expected log-likelihood (Eq (8)) was performed using LocalSolver with a running time of 600 s. The EM algorithm was iterated until the values of *μ*^(*l*)^(*kδ*) or *η*^(*l*)^(*kδ*) converged, and the convergence was examined using the following criteria:

$$\begin{array}{c} \sqrt{\frac{\sum_{k}{\left(\mu^{\left(l\right)}\left(k\delta \right)-\mu^{\left(l-1\right)}\left(k\delta \right)\right)}^{2}}{\sum_{k}\mu^{\left(l-1\right)}{\left(k\delta \right)}^{2}}}<0.001\text{,}\;\text{or}\;\sqrt{\frac{\sum_{k}{\left(\eta^{\left(l\right)}\left(k\delta \right)-\eta^{\left(l-1\right)}\left(k\delta \right)\right)}^{2}}{\sum_{k}\eta^{\left(l-1\right)}{\left(k\delta \right)}^{2}}}<0.001. \end{array}$$ (11)

However, the EM calculation does not meet the convergence criteria for the point processes of SoftBank, SONY, and MitsubishiUFJ, presumably because of their large sizes. We examined the validity of our estimation for these stocks, as shown in S.2 of S1 Text. For both numerical calculations, we used a computer with two CPUs (18-Core, 2.20 GHz, Inter Xeon Gold processors) and 192 GB of memory.

## Results

### Overview of the transaction frequency

**Fig 3:**
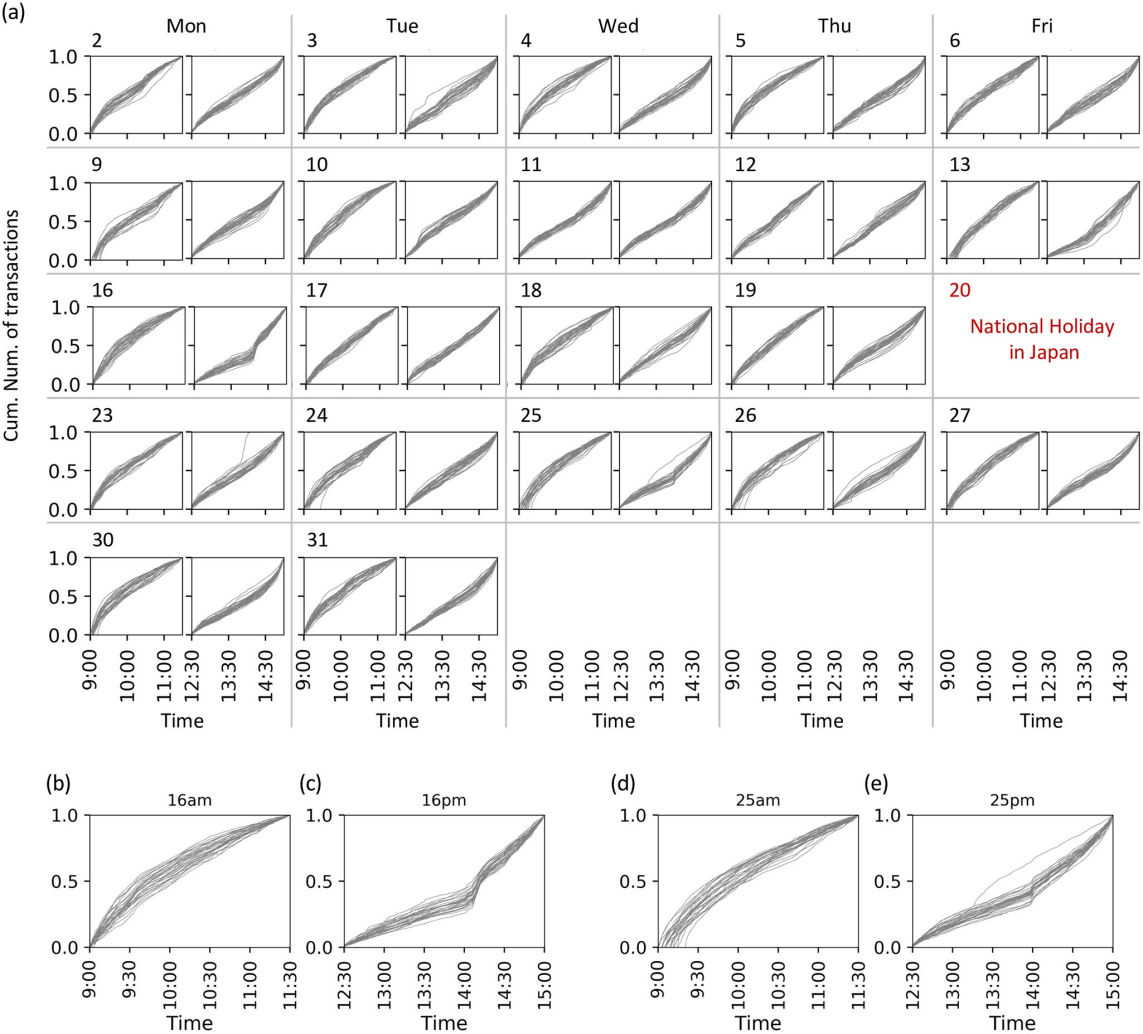
Cumulative number of transactions. (a) Each panel corresponds to each date and session, as shown in the upper left of the panel. Panels (b), (c), (d), and (e) show the results in the morning session on March 16, afternoon session on March 16, morning session on March 25, and afternoon session on March 25, respectively, in an enlarged view. For each panel, the horizontal axis shows time *t*, and the vertical axis shows the cumulative number of transactions at *t* normalized by the total number of transactions in each trading session. Each plot corresponds to a stock.

In this section, we provide an overview of the transaction frequency during the analyzed period. Fig 3 plots the cumulative number of transactions against time for each stock, session, and date. A steep increase in the cumulative number indicated that many transactions occurred at that time. We found a prominent increase for multiple stocks after 2 pm on March 16 and a steep increase between 1:30 and 2:30 pm on March 25. On March 16, the Bank of Japan (BOJ) Policy Board’s monetary policy meeting was held from 0:00 pm to 1:59 pm [35–37]. This meeting was held earlier than what was originally scheduled, in response to the financial instability caused by COVID-19 spreading. The BOJ announced the enhancement of monetary easing after 2 pm on that day. Therefore, the rapid increase in the cumulative number of transactions after 2 pm on March 16 indicates investors’ responses to news regarding BOJ announcements. Similarly, the burst of transactions on March 25 was presumably caused by news in the US. At approximately 2 pm on that day in Japan, which is in the early morning in the US, the agreement between Senate leaders and the Trump administration was reported. The agreement was regarding a stimulus package to rescue the economy during the COVID-19 pandemic [38, 39].

### Estimated intensity

**Fig 4:**
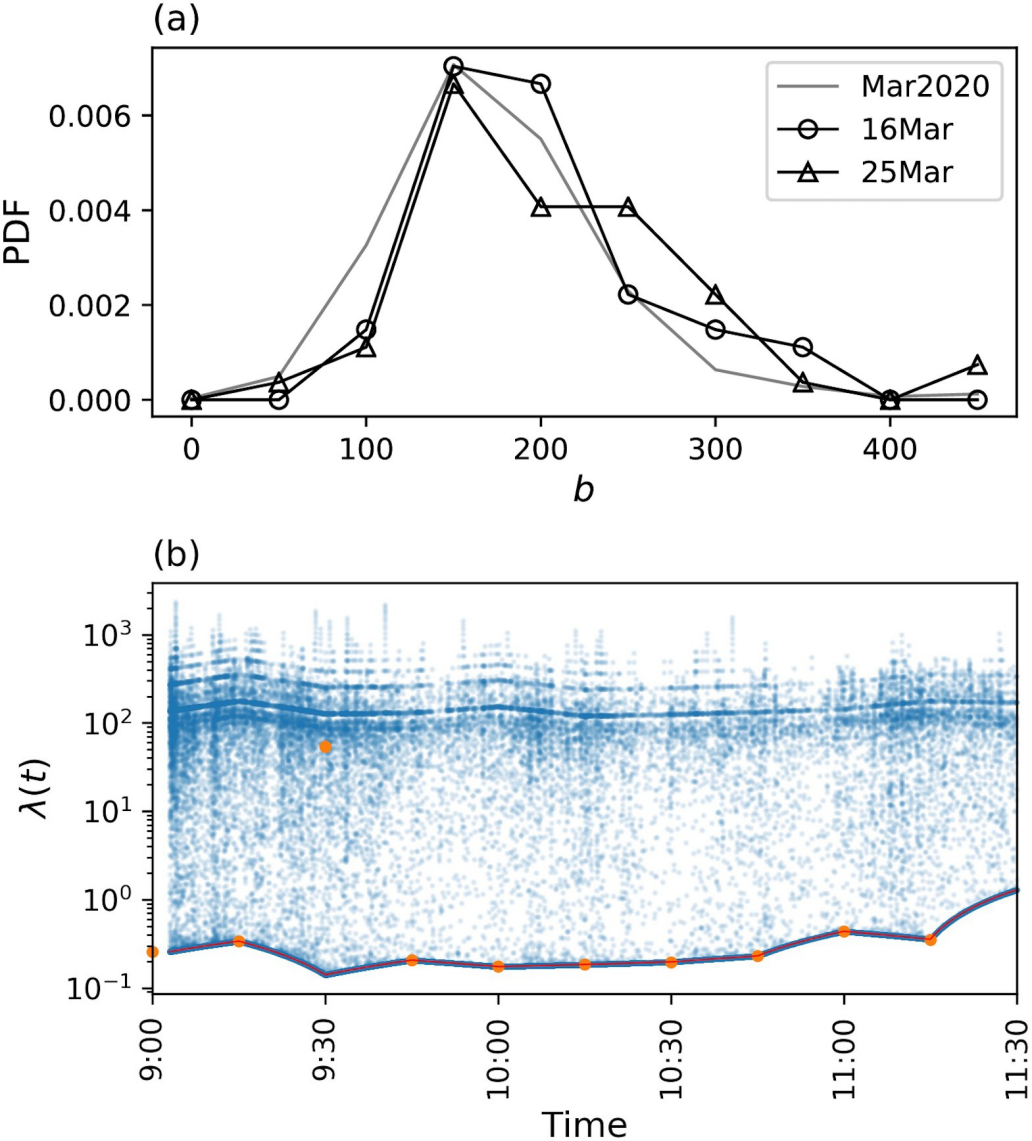
Exponent in memory kernel and intensity function. (a) Estimated value of exponent *b* in memory kernel. Each plot shows the probability density function (PDF) of the values of *b* over all stocks. PDFs for March 16 and 25 are shown by circles and triangles, respectively, and PDF over all dates in March is shown in grey line. (b) An example of intensity function λ(*t*) calculated by the estimated *μ*(*t*), *η*(*t*), and *b* (morning session on March 16, SoftBank). The horizontal and vertical axes show the time *t*_*i*_ of the *i*th event and intensity λ(*t*_*i*_), respectively. The vertical axis is logarithmically scaled. The estimated *μ*(*t*) is plotted in red. Orange circles exhibit (*kδ*, λ(*kδ*)) (*k* = 0, 1, …, 10, *δ* = 100 s).

We modeled the transaction frequency using the Hawkes process (Eq (2)) and estimated the values of the parameters associated with the model for each day, trading session, and stock. The exponent *b* of the memory kernel represents the extent to which the effect of a single transaction on exciting others remains. The estimated values of *b* are considerably large (Fig 4(a)) [40], indicating that the excitation effect can only last for a short period. Fig 4(b) shows an example of the intensity function λ(*t*) modeled by Eq (2) using the estimated values of *b*, *μ*(*t*), and *η*(*t*). Although the value of λ(*t*) was similar to that of the background intensity *μ*(*t*), it discretely increased as a transaction occurred and rapidly declined to return to the level of the baseline *μ*(*t*) due to a large *b*.

### Estimated background intensity and branching ratio

**Fig 5:**
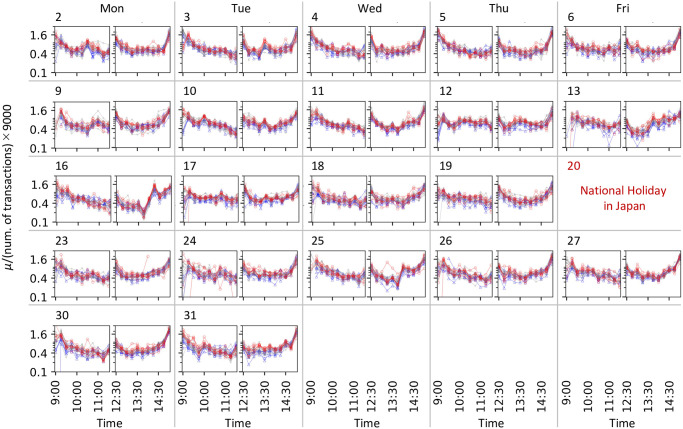
Estimated *μ*(*t*). Each panel corresponds to each date and session, as shown in the upper left of the panel, where each column of the array corresponds to a weekday. For each panel, each plot corresponds to a stock. The horizontal and vertical axes show the time *t* and *μ*(*t*) normalized by the number of transactions for each stock in each session, respectively. For calculating the normalized *μ*(*t*), *μ*(*t*) is divided by the total number of transactions and multiplied by 9,000, considering the length of each session (9,000 s). The normalized *μ*(*t*) of the stocks in clusters A and B are plotted in red and blue, respectively, where these clusters are determined in Fig 9(a). The normalized *μ*(*t*) of the other stocks are plotted in grey.

**Fig 6:**
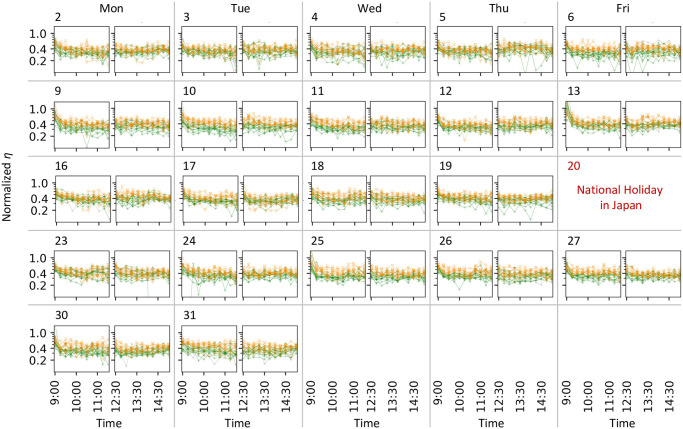
Estimated *η*(*t*). Panels are arranged in the same manner as those in Fig 5. For each panel, each plot corresponds to a stock. The horizontal and vertical axes show time *t* and *η*(*t*), respectively. *η*(*t*) of stocks in clusters C and D are shown in orange and green, respectively, where these clusters are determined in Fig 9(b). The values of *η*(*t*) of the other stocks are plotted in grey.

**Fig 7:**
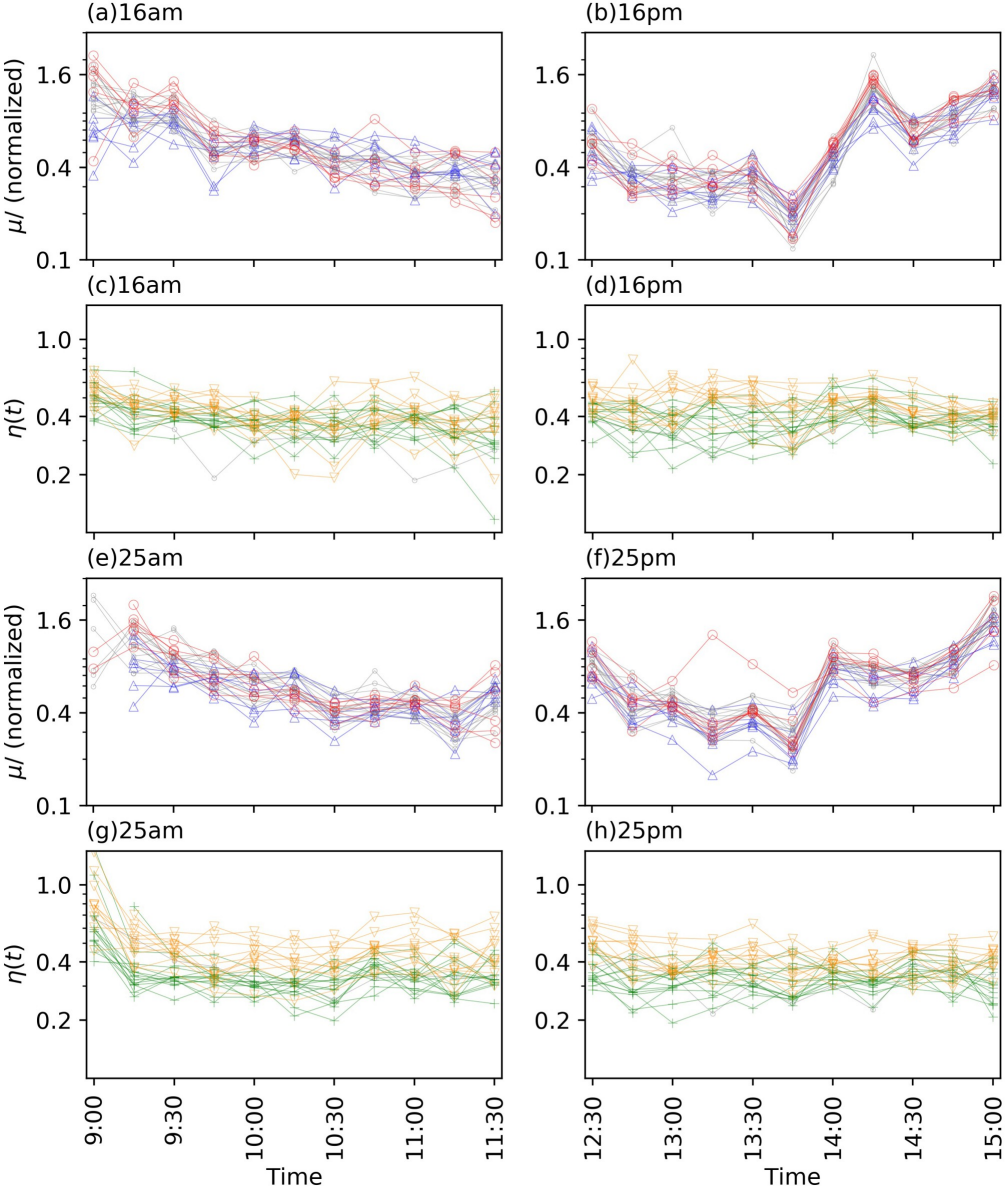
Enlarged views of estimated *μ*(*t*) and *η*(*t*) shown in Figs 5 and 6. Panels (a, c, e, g) and (b,d,f,h) show the results in the morning and afternoon sessions, respectively. Panels (a,b,e,f) and (c,d,g,h) show the results on March 16 and 25, respectively. For each panel, the normalized *μ*(*t*) and *η*(*t*) are plotted similar to in Figs 5 and 6, respectively.

Subsequently, we investigated the temporal changes in the background intensity *μ*(*t*) and branching ratio *η*(*t*). *μ*(*t*) is the effect of external events on the transaction frequency, such as news and accidents, or intraday patterns in the transaction frequency. In Figs 5, 7(a), 7(b), 7(e) and 7(f), we demonstrated *μ*(*t*) normalized by the number of transactions for each stock in each session. The normalized *μ*(*t*) can provide a comparison between stocks regarding the transition in the strength of external factors, whereas the absolute values of *μ*(*t*) reflect the magnitude of the transaction frequency for each stock. The normalized *μ*(*t*) exhibits a common tendency over the analyzed days and stocks (Fig 5), which is large at the beginning of each trading session and at the end of the afternoon session. However, after 2 pm on March 16 when the news of monetary easing was announced and at approximately 2 pm on March 25 when that of the stimulus package in the US was reported, the normalized *μ*(*t*) significantly increased and exhibited great values, while such increases cannot be observed in the afternoon sessions on the other days that were analyzed (Fig 7(a), 7(b), 7(e) and 7(f)). By contrast, we do not observe such an extreme increase in the value of *η*(*t*) at that time (Figs 6, 7(c), 7(d), 7(g) and 7(h)). The branching ratio *η*(*t*) represents the number of transactions that a single transaction at time *t* can generate (i.e., the strength of the endogenous factors generating transactions). Therefore, the estimated *μ*(*t*) and *η*(*t*) suggest that the transaction bursts on March 16 and 25 were caused mainly by the exogenous factor of news arrival. Another finding is that the value of *η*(*t*) is less than 1. If *η*(*t*) is greater than 1, it suggests an uncontrolled state in which the number of transactions shows an explosive increase. Thus, from the viewpoint of the self-excitation of transactions, the market can be regarded as stable, even on March 16 or 25.

**Fig 8:**
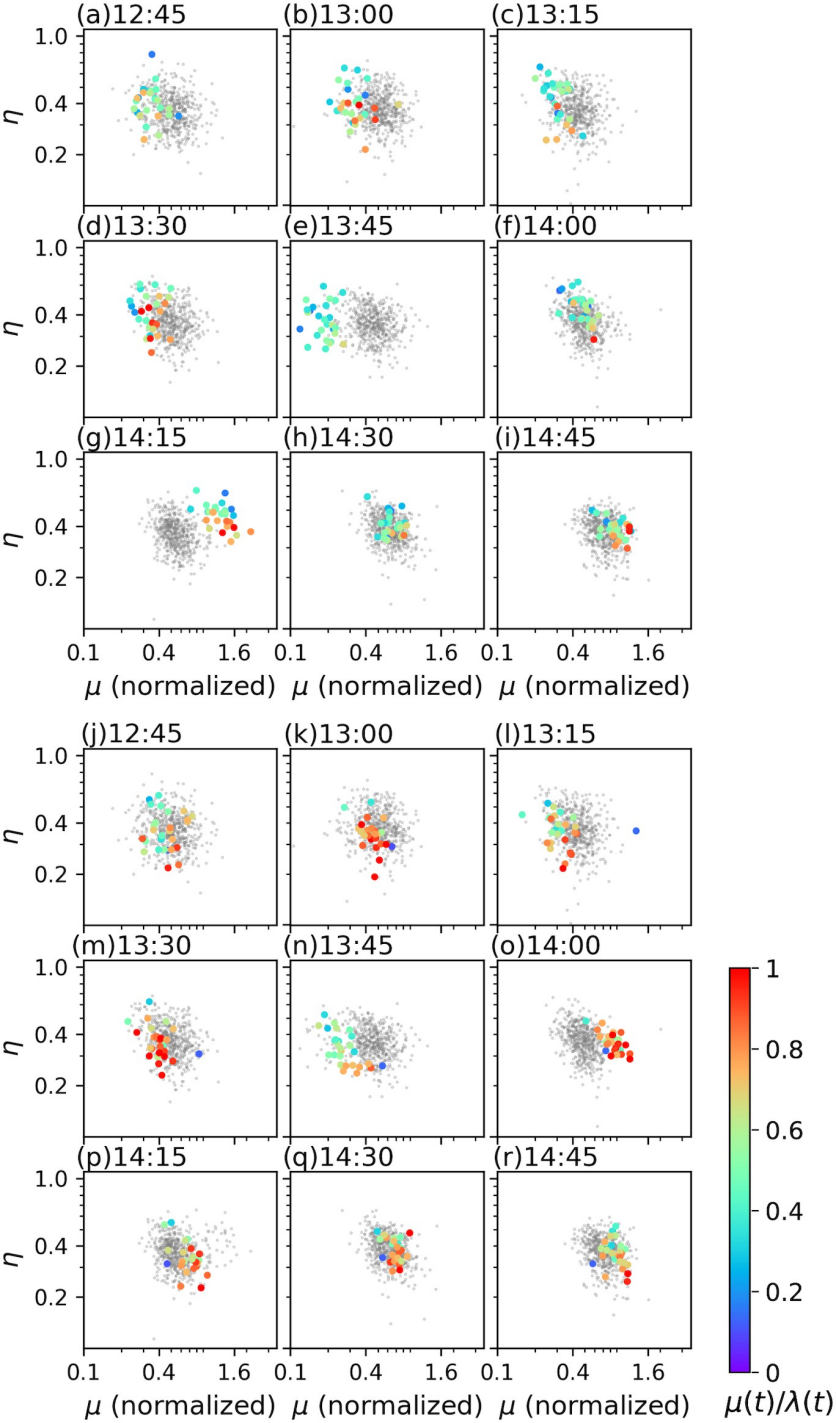
*μ*(*t*) and *η*(*t*) in the afternoon session on March 16 (a-i) and March 25 (j-r). Each panel corresponds to the time shown in the upper-left corner of the panel. The horizontal and vertical axes show *μ*(*t*) normalized by the number of transactions and *η*(*t*), respectively. Grey circles show normalized *μ*(*t*) and *η*(*t*) for all stocks in the afternoon session on all dates in March. Only the plots for all stocks in the afternoon session on March 16 or 25 are highlighted in color based on the value of *μ*(*t*)/λ(*t*), in panels (a-i) or panels (j-r), respectively. λ(*t*) is calculated as the number of transactions in [*t* − *δ*/2, *t* + *δ*/2] divided by *δ*.

We investigated the nature of the transaction frequency in the afternoon session on March 16 by characterizing it with a combination of *μ*(*t*) and *η*(*t*) and found an interesting feature at approximately 2 pm. Fig 8(a)–8(i) show a pair of *μ*(*t*) normalized by the number of transactions and *η*(*t*). The plots of *μ*(*t*) and *η*(*t*) are shown for all stocks each time in the afternoon session on all days that were analyzed, where only the plots for March 16 are highlighted. The plots for March 16 are colored based on the value of *μ*(*t*)/λ(*t*), which represents the contribution of the background intensity to the transaction frequency relative to the intensity at that time. The intensity λ(*t*) at *t* is approximated by the number of transactions in [*t* − *δ*/2, *t* + *δ*/2] divided by *δ* by considering the discontinuity of the intensity function (Eq (2)). At 13 : 30 and 13 : 45 (Fig 8(d) and 8(e)), the normalized *μ*(*t*) for the analyzed stocks on March 16 showed smaller values than those on the other days. In addition, *μ*(*t*)/λ(*t*) at 13 : 45 is small, suggesting that exogenous factors suppress transactions. By contrast, at 14 : 15, the normalized *μ*(*t*) becomes large. Additionally, *η*(*t*) at that time was larger than that on the other days (Fig 8(g)). Therefore, the transition of *μ*(*t*) and *η*(*t*) suggests a situation in which transactions caused by external factors were suppressed during the BOJ meeting and frequently occurred after the post-meeting announcement, which was accompanied by an increase in self-excitation among transactions.

The temporal changes in *μ*(*t*) on March 25 exhibited the suppression and the great response of transactions against the news of the stimulus package in the US. Fig 8(j)–8(r) show a pair of *μ*(*t*) and *η*(*t*) in the afternoon session on March 25 similar to that in Fig 8(a)–8(i). At 13:45, the normalized *μ*(*t*) or the values of *μ*(*t*) relative to λ(*t*) of most issues are considerably small. These values turned out to be large at 14:00. In contrast to the case of March 16, the values of *η*(*t*) did not increase accompanied by *μ*(*t*) on March 25. Therefore, the endogenous factors made little contribution to the transaction frequency on March 25, while the great response against the external news was observed on that day as well as on March 16.

### Clustering of stocks

**Fig 9:**
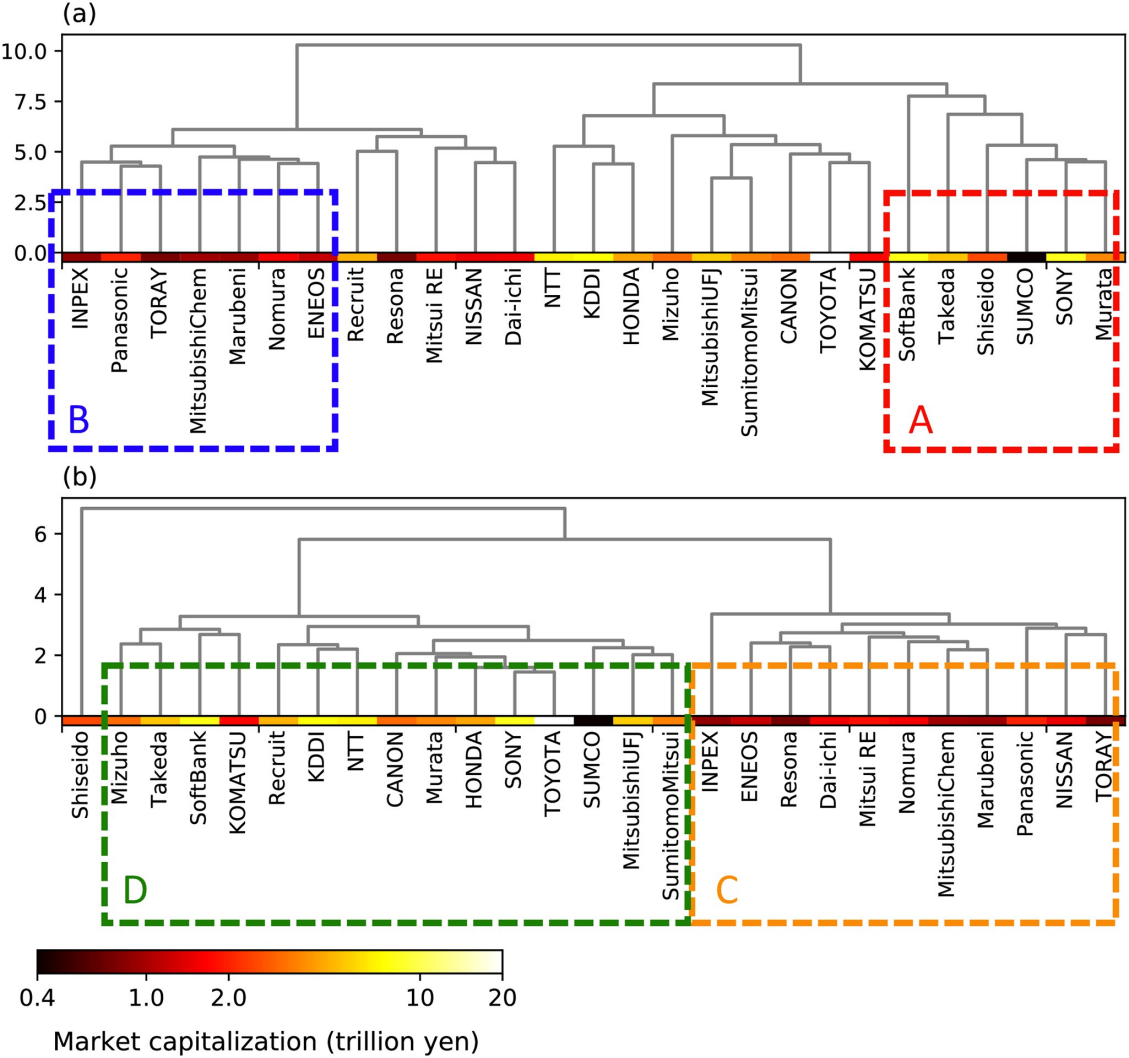
Dendrogram of stocks constructed using Ward’s method based on (a) the distance $d(v_{k}^{\mu },v_{l}^{\mu })$ associated with *μ*(*t*) and (b) the distance $d(v_{k}^{\eta },v_{l}^{\eta })$ associated with *η*(*t*). The color of each stock represents its market capitalization as of the end of March 2020, which is represented in a logarithmic scale, as shown in the legend. *μ*(*t*) and *η*(*t*) of stocks in clusters A, B, C and D in this figure are highlighted in red, blue, orange, and green in Figs 5 and 6.

We further analyzed the similarity relationship between stocks from the viewpoint of temporal changes in the background intensity and branching ratio. A dendrogram of the stocks was constructed (Fig 9(a)) by defining the distance between them based on their normalized *μ*(*t*) as follows: Let $v_{s,d,\text{am}}^{\mu }$ ($v_{s,d,\text{pm}}^{\mu }$) be the 11-dimensional vector of the estimated values of *μ*(*kδ*) (*k* = 0, 1, …, 10) for stock *s* during the morning (afternoon) session on day *d*. We define the normalized vector of *μ*(*t*), ${\tilde{v}}_{s,d,\text{am}}^{\mu }$ (${\tilde{v}}_{s,d,\text{pm}}^{\mu }$), by dividing each element of $v_{k,d,\text{am}}^{\mu }$ ($v_{k,d,\text{pm}}^{\mu }$) by the number of transactions for each stock in the morning (afternoon) session of that day. Thereafter, we construct a 462-dimensional vector

$$\begin{array}{c} v_{s}^{\mu }=\left({\tilde{v}}_{s,\;\text{2Mar},\;\text{am}}^{\mu },\;{\tilde{v}}_{s,\;\text{2Mar},\;\text{pm}}^{\mu },\;...,\;{\tilde{v}}_{s,\;\text{31Mar},\;\text{am}}^{\mu },\;{\tilde{v}}_{s,\;\text{31Mar},\;\text{pm}}^{\mu }\right), \end{array}$$

consisting of ${\tilde{v}}_{k,d,\text{am}}^{\mu }$ and ${\tilde{v}}_{k,d,\text{pm}}^{\mu }$ of all dates in March, where 462 = 11 × 2 (sessions) × 21 (days). Vector $v_{s}^{\mu }$ represents the characteristics of stock *s* regarding the effects of external factors on transactions. The distance between stocks *s* and *s*^′^, $d(v_{s}^{\mu },v_{s^{\prime }}^{\mu })$, is defined by the Euclidean norm as:

$$\begin{array}{c} d\left(v_{s}^{\mu },v_{s^{\prime }}^{\mu }\right)=\sqrt{\sum_{i=1}^{66}{\left(v_{s,i}^{\mu }-v_{s^{\prime },i}^{\mu }\right)}^{2}}, \end{array}$$

where $v_{s,i}^{\mu }$ is the *i*th entry of $v_{s}^{\mu }$. We constructed a dendrogram of these stocks (Fig 9(b)) based on the distance $d(v_{s}^{\eta },v_{s^{\prime }}^{\eta })$ defined with respect to *η*(*t*) of stocks *s* and *s*^′^ similar as $d(v_{s}^{\mu },v_{s^{\prime }}^{\mu })$. However, we did not normalize the value of *η*(*kδ*) to the number of transactions in the construction of the characteristic vector $v_{s}^{\eta }$.

Dendrograms based on the distance considering *μ*(*t*) and *η*(*t*) exhibit different similarity relationships between stocks and the characteristics of stocks from the viewpoint of the exogenous and endogenous factors generating their transactions. Fig 9(a) illustrates the dendrogram constructed based on the normalized *μ*(*t*). A cluster of stocks indicates that their transactions are similarly affected by external news throughout March. Stocks in specific clusters, such as clusters A and B in Fig 9(a), have distinct features regarding the temporal changes in *μ*(*t*). In Fig 5, the values of the normalized *μ*(*t*) for stocks included in cluster A are plotted in red, while the stocks in cluster B are plotted in blue. *μ*(*t*) of the stocks in cluster A (red) took significantly high values after 2 pm on March 16 or at 2 pm on March 25, when *μ*(*t*) of most stocks rapidly increased. In contrast, *μ*(*t*) of stocks in cluster B show weaker responses than those in cluster A at the peaks of *μ*(*t*) on March 16 and 25. When the similarity among stocks was evaluated based on their *η*(*t*) values, the stocks were differently clustered from those that have been discussed (Fig 9(b)). Fig 6 illustrates that stocks in the two clusters regarding the similarity in *η*(*t*) are also colored. Stocks highlighted in orange and green are in clusters C and D in Fig 9(b), respectively. Stocks in cluster C consistently exhibit higher values of *η*(*t*) than those in cluster D throughout the month, suggesting a robust characteristic where the former stocks are more influenced by self-excitation between transactions than the latter.

**Fig 10:**
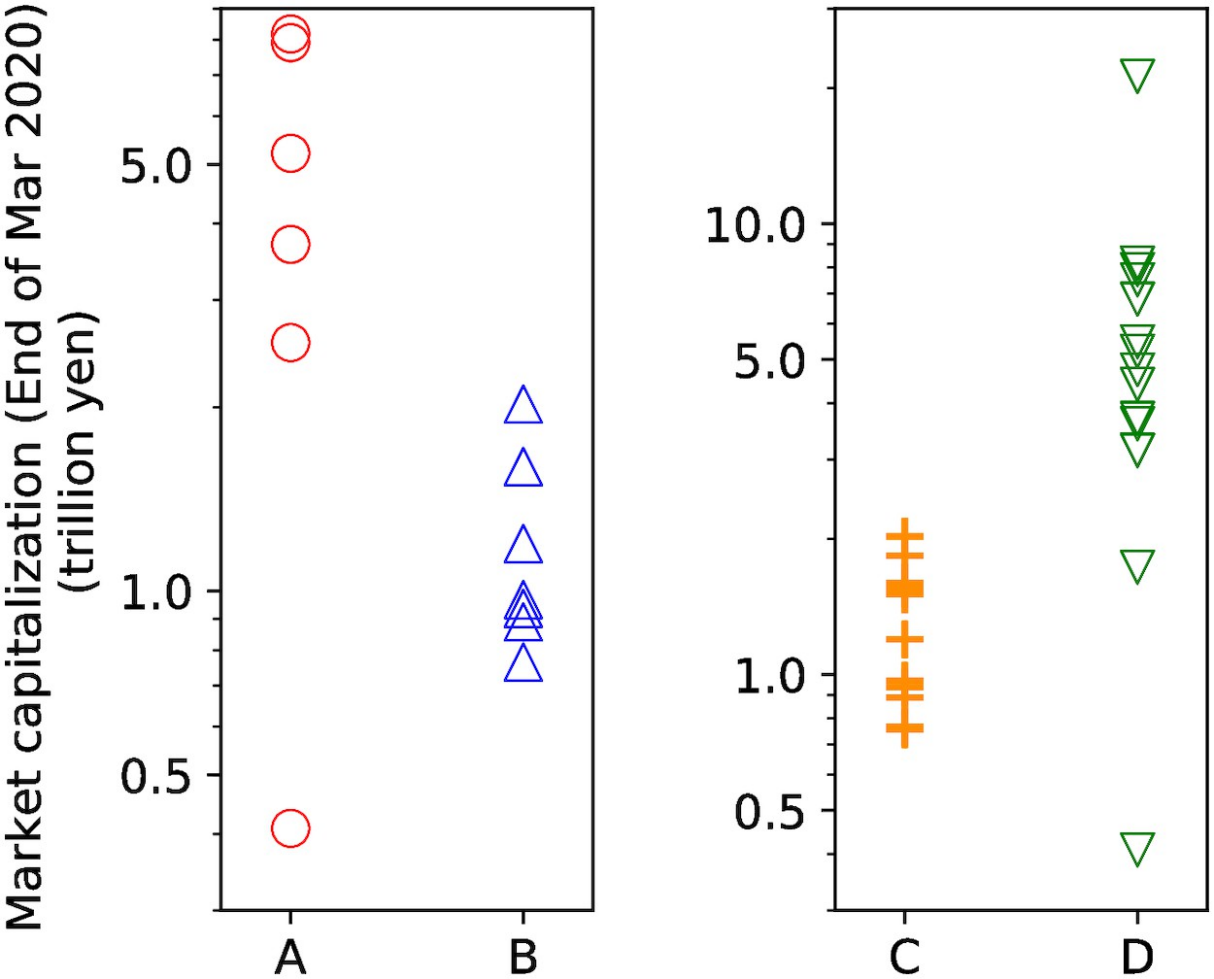
Market capitalization of stocks included in clusters determined in Fig 9. The horizontal axis shows the names of the clusters, and the vertical axis shows the market capitalization. Each plot corresponds to each stock in the cluster.

Furthermore, the characteristics of stocks quantified based on *μ*(*t*) and *η*(*t*) are related to the firms’ sizes. Fig 10 illustrates the market capitalization as of the end of March 2020 of stocks included in clusters A, B, C, and D. Additionally, the color shown above each stock name in the dendrograms (Fig 9) represents its market capitalization. Stocks in cluster A, which greatly responded to the external factors on March 16 and 25, tend to have higher market capitalization than those in cluster B. Regarding the endogenous factors, most stocks in cluster C, showing high values of *η*(*t*), have lower market capitalization than those in cluster D. Additionally, five stocks out of six in cluster A are included in cluster D, indicating that stocks with high market capitalization greatly respond to external news, while their interaction between transactions is weak. The tendencies in the transitions of *μ*(*t*) and *η*(*t*) for each cluster indicate that stocks can be characterized based on the extent to which their transactions are generated by exogenous and endogenous factors, respectively.

## Discussion

In this study, we attempted to infer the temporal changes in the factors motivating investors to perform transactions from the history of the transaction frequency. We adopted an analysis based on the Hawkes process, in which events are assumed to occur due to the effect of self-excitation between events (endogenous factors) and factors other than the excitation relationship, such as external shocks or a general trend in event frequency (exogenous factors). We regarded a transaction as an event of the Hawkes process and analyzed the transaction data of TSE in March 2020, when the market was in the COVID-19 financial turmoil. The estimated strengths of the endogenous and exogenous factors for each stock’s transactions temporally changed, providing data-driven information on the background of the transaction frequency.

Our results show the investors’ responses to the arrival of news and the intensified interactions between transactions after an announcement by the financial authority on March 16. The estimated background intensity (i.e., the strength of the exogenous factor) was small as the meeting of BOJ continued; however, this proved to be large after the announcement of financial measurement after the meeting. This temporal change suggests that investors waited for the meeting’s decision by suppressing their transactions and greatly responded after the announcement. With respect to BOJ’s monetary policy meetings, the timing of the decision announcement is not fixed [41, 42]. The duration of the meeting can be considered as an external factor affecting investors’ transactions. Moreover, we obtained that the endogenous factor was large after the announcement, accompanied by a large exogenous factor, indicating that transactions caused by external news further excited those that followed. Therefore, a burst of transactions after the authority’s announcement can be regarded as produced by a mixture of the effect of external news and interaction between transactions, presumably caused by investors who were sensitive to government measures. The suppression and rapid increase in the strength of exogenous factors were also observed when the news of the stimulus package in the US was reported on March 25. However, in this case, the endogenous factors did not significantly affect transactions accompanied by the exogenous ones, which is different from the case of March 16. This gap implies that the forces generating transactions could differently change according to the type of news. We expect that we may find patterns in the combination of temporal changes in these factors by further investigating the strength of endogenous and exogenous factors on various occasions, leading to the quantification of the market state.

Furthermore, we carried out clustering of stocks from the viewpoint of their resemblance in the exogenous and endogenous factors generating transactions and found some characteristics of the stocks. On days when authorities’ responses to economic disruptions in the COVID-19 pandemic were reported, the background intensity for stocks in a cluster that significantly responded after these announcements was notable. Additionally, we identified clusters of stocks where the branching ratio was consistently high or low throughout the month. These results indicate stocks that are highly sensitive to specific external information or whose transactions strongly trigger the subsequent ones. Furthermore, the characteristics of stocks in relation to the strength of the exogenous and endogenous factors were associated with their market capitalization. We found a tendency where stocks with high market capitalization, representing larger firms, greatly respond to external news, while the interaction between their transactions is relatively weaker.

This comparison between the time-varying characteristics of the transaction frequency for various stocks was achieved by the analyses of more than a thousand point processes, based on a simple Hawkes model, except for the consideration of their temporal changes. Previous studies have suggested more complex intensity functions for Hawkes models than for the original models. For instance, modified intensity functions include additional terms to distinguish among various types of exogenous factors [5, 8, 43]. Furthermore, studies on financial point processes often support and adopt power-law memory kernels that represent long-term memory in financial phenomena [5–8, 44–48]. Regarding power-law memory kernels, many studies have assumed the function of the power law with an exponential cut-off that can be approximated by the sum of exponential functions. The number of exponential functions required to represent the memory kernels is often set as a parameter to be calibrated, and the resulting number varied in previous studies [6, 45]. Although these careful evaluations provide a precise estimation of endogenous and factors, they require the calibration of many parameters for each point process, and the number of parameters can differ among financial point processes. Therefore, we assumed a simple Hawkes model with an exponential memory kernel to reduce the computational cost of our analysis by referring to previous studies that examined numerous point processes as well as our study [49]. We expected that selecting a simple Hawkes model is suitable for our research objective of outlining the entire picture of the transaction frequency in a market.

Furthermore, we considered the temporal continuity of the strength of self-excitation and background intensity more carefully than the previous study [8]. For example, the strength of self-excitation can reflect the investors’ mindset and should continuously change. By considering the mutual dependence of these factors among different time points, we simultaneously estimated those values by solving an optimization problem. Thus, the results of our estimation are feasible in the sense that they reflect the temporal continuity of the strength of endogenous and exogenous factors.

In future work, we may find another relationship among stocks that generates patterns of transaction frequency in a market by examining the mutual excitation between transactions for different stocks. Our results on the transaction frequency show simultaneous increases in the transaction intensity for multiple stocks, suggesting a correlation structure among these stocks. A set of the transaction frequency for multiple stocks can be modeled using a multivariate Hawkes process [5, 50, 51]. Using this model, we can estimate the extent to which a stock’s transaction excites the transactions of another stock (i.e., the mutual excitation relationship between stocks). The calculation of the mutual excitation among many stocks is computationally expensive and requires the development of an efficient algorithm. However, an evaluation of their mutual excitation relationships should reveal an important aspect in the ecology of a market.

Future research should conduct a more comprehensive analysis by including data from months adjacent to the study period. This approach will allow us to examine whether our findings in the present study are specific to unique circumstances in March 2020. Specifically, by extending the analyzed period, we can compare the characteristics of the impact of investors’ sentiment or external news on transactions between the period of financial instability during the pandemic and other periods. Moreover, extending the analysis over a longer period will provide deeper insights into market behaviors under varying economic conditions.

## Supporting information

S1 Text Supplementary information for exogenous and endogenous factors for stock transactions: A Hawkes process analysis of the Tokyo Stock Exchange during COVID-19 pandemic. In this paper, we presented a detailed calculation related to the analyses and validity of our estimates.
(PDF)
