# Supplementary material for: Exogenous and endogenous factors affecting stock market transactions: A Hawkes process analysis of the Tokyo Stock Exchange during the COVID-19 pandemic
Source: PLoS One. 2024 Apr 17;19(4):e0301462. doi: 10.1371/journal.pone.0301462 (PMC11023603; doi:10.1371/journal.pone.0301462)
Supplement: S1 Text — In this paper, we presented a detailed calculation related to the analyses and validity of our estimates. (PDF) [file pone.0301462.s001.pdf]

Supplementary information for Exogenous and Endogenous Factors for Stock Transactions: A Hawkes Process Analysis of the Tokyo Stock Exchange during COVID-19 Pandemic

Mariko I. Ito, Yudai Honma, Takaaki Ohnishi,
Tsutomu Watanabe, Kazuyuki Aihara

S.1 Detailed calculation in E-step

In this section, we present the derivation of the expected log-likelihood over possible branching structures, $\mathrm{E}_{Z|\{t_i\},\theta^{(l-1)}}\left[\log L(\theta|\{t_i\},Z)\right] \left(=: Q\left(\theta|\{t_i\},\theta^{(l-1)}\right)\right)$, in the E-step of the EM algorithm described in the Methods section of the main text.

S.1.1 Log-likelihood of parameters for Hawkes process

First, we show the log-likelihood of the parameters for the Hawkes process. Here, we assume a point process $\{t_i\}$ in the interval $[0,T]$, where the total number of events is n_T.

Let $\lambda(t)$ be the intensity function of the point process; that is:

$$\Pr[\text{an event occurs in } [t,t+\Delta]] \approx \lambda(t)\Delta, \tag{S.1}$$

where Δ is infinitesimally small such that at most one event occurs in $[t,t+\Delta]$. The probability $\Pr[\{t_i\}]$ of observing a point process $\{t_i\}$ can be approximated as

$$\Pr[\{t_i\}] \approx \Delta^{n_T} \prod_i \lambda(t_i) \exp\left(-\int_0^T \lambda(t)\mathrm{d}t\right), \tag{S.2}$$

where $\prod_i \lambda(t_i)\exp\left(-\int_0^T \lambda(t)\mathrm{d}t\right)$ denotes the probability density function (PDF) of the point process.

Hereafter, we assume a Hawkes process with intensity function $\lambda(t)$:

$$\lambda(t) = \mu + \sum_{i:t_i<t} \eta h(t-t_i), \tag{S.3}$$

where $h(t) = b\exp(-bt)$. Let θ be a set of the parameters μ, η, and b in the Hawkes model.

The probability $\Pr[\{t_i\}]$ of observing point process $\{t_i\}$ can regarded as the likelihood function $L(\theta)$ of θ provided $\{t_i\}$. Therefore, the likelihood of θ for the Hawkes process with intensity provided by Eq. (S.3) can be calculated as

$$L(\theta) \propto \prod_{i=1}^{n_T}\Bigg(\mu + \sum_{j;t_j<t_i} \eta h(t_i - t_j)\Bigg) \exp\left(-\int_0^T \Bigg(\mu + \sum_{i:t_i<t} \eta h(t - t_i)\Bigg) \mathrm{d}t\right). \tag{S.4}$$

Therefore, the log-likelihood function of θ is denoted by:

$$\log L(\theta) = \log\left[\prod_{i=1}^{n_T}\Bigg(\mu + \sum_{j;t_j<t_i} \eta h(t_i - t_j)\Bigg) \exp\left(-\int_0^T \Bigg(\mu + \sum_{i:t_i<t} \eta h(t - t_i)\Bigg) \mathrm{d}t\right)\right] + C \tag{S.5}$$

$$= \sum_{i=1}^{n_T} \log\Bigg(\mu + \sum_{j;t_j<t_i} \eta h(t_i - t_j)\Bigg) - \int_0^T \Bigg(\mu + \sum_{i:t_i<t} \eta h(t - t_i)\Bigg) \mathrm{d}t + C \tag{S.6}$$

$$= \sum_{i=1}^{n_T} \log\Bigg(\mu + \sum_{j;t_j<t_i} \eta h(t_i - t_j)\Bigg) - \mu T - \eta \sum_{i=1}^{n_T} \int_0^{T-t_i} h(s)\mathrm{d}s + C, \tag{S.7}$$

where C is a constant and the maximum likelihood estimate $\hat{\theta}$ is

$$\hat{\theta} = \underset{\theta}{\mathrm{argmax}}\left[\sum_{i=1}^{n_T} \log\Bigg(\mu + \sum_{j;t_j<t_i} \eta h(t_i - t_j)\Bigg) - \mu T - \eta \sum_{i=1}^{n_T} \int_0^{T-t_i} h(s)\mathrm{d}s\right]. \tag{S.8}$$

If μ and η change temporally, the intensity function is expressed by:

$$\lambda(t) = \mu(t) + \sum_{i:t_i<t} \eta(t) h(t - t_i), \tag{S.9}$$

where $h(t) = b\exp(-bt)$ and the log-likelihood estimate $\hat{\theta}$ is derived as

$$\begin{aligned}\hat{\theta} &= \underset{\theta}{\mathrm{argmax}}\left[\sum_{i=1}^{n_T} \log\Bigg(\mu_i + \sum_{j;t_j<t_i} \eta_j h(t_i - t_j)\Bigg) - \int_0^T \Bigg(\mu(t) + \sum_{i:t_i<t} \eta_i h(t - t_i)\Bigg) \mathrm{d}t\right] \\ &= \underset{\theta}{\mathrm{argmax}}\Bigg[\sum_{i=1}^{n_T} \log\Bigg(\mu_i + \sum_{j;t_j<t_i} \eta_j b\exp\big(-b(t_i - t_j)\big)\Bigg) \\ &\qquad - \int_0^T \mu(t)\mathrm{d}t - \sum_{i=1}^{n_T} \eta_i \Big\{1 - \exp\big(-b(T - t_i)\big)\Big\}\Bigg],\end{aligned} \tag{S.10}$$

where μ_i and η_i respectively mean $\mu(t_i)$ and $\eta(t_i)$.

S.1.2 Expected log-likelihood of parameters over possible branching structure

For a point process $w := \{t_i\}$ of transactions, we considered the lineage relationship between the transactions, called the branching structure Z:

$$Z_{i,j} = \begin{cases} 1, & \text{if } i \neq j \text{ and event } x_i \text{ is generated by event } x_j, \\ 1, & \text{if } i = j \text{ and event } x_i \text{ is exogenously generated}, \\ 0, & \text{otherwise} \end{cases} \tag{S.11}$$

as described in the Methods section of the main text. Here, x_i denotes an event that occurs at time t_i. Furthermore, we consider the probabilities $\pi_{i,j}$ $(i \neq j)$ and $\pi_{i,i}$ of x_i being endogenously generated by x_j and being exogenously generated at time t_i, respectively $(\sum_{j<i} p_{i,j} + p_{i,i} = 1)$.

The Hawkes process, defined by the intensity function in Eq. (S.3) is shown to be equivalent to a branching process in which an event is exogenously generated at rate μ and each event further generates another one or reproduces an offspring at rate $\eta h(t - t_i)$ at time t [1]. Considering this relationship between Hawkes and branching processes, π can be calculated as follows:

$$\pi_{i,i} = \frac{\mu_i}{\lambda(t_i)}, \tag{S.12}$$

$$\pi_{i,j} = \frac{\eta_j h(t_i - t_j)}{\lambda(t_i)}. \tag{S.13}$$

Based on the relationship between Hawkes and branching processes, the probability of a transaction occurring in an infinitesimally small interval $[t_i,\ t_i + \Delta]$ is denoted by

$$\Pr\left[\text{an event occurs in } [t_i, t_i + \Delta] | Z\right] \approx \begin{cases} \Delta\mu(t_i), & \text{if } Z_{i,i} = 1, \\ \Delta\eta(t_j)h(t_i - t_j), & \text{if } {}^{\exists}j(\neq i) \text{ s.t. } Z_{i,j} = 1. \end{cases} \tag{S.14}$$

Note that for event x_i, there is a single event x_j such that $Z_{i,j} = 1$ if $Z_{i,i} = 0$. Therefore, the probability of observing point process w with branching structure Z can be written as

$$\Delta^{n_T} \prod_{i=1}^{n_T} X_i \cdot \exp\left(-\int_0^T \lambda(t)\mathrm{d}t\right), \tag{S.15}$$

where

$$X_i := \begin{cases} \mu(t_i), \text{ if } Z_{i,i} = 1, \\ \eta(t_j)h(t_i - t_j), \text{ if } {}^{\exists}j(\neq i) \text{ s.t. } Z_{i,j} = 1 \end{cases} \tag{S.16}$$

by Eq. (S.2).

The expected log-likelihood $\mathrm{E}_Z\left[\log L(\theta|w, Z)\right]$ over the possible branching structure Z is calculated as follows:

$$
\begin{aligned}
&\mathrm{E}_Z\left[\log L(\theta|w, Z)\right] && \text{(S.17)}\\
&= \mathrm{E}_Z\left[\log\left(\Delta^{n_T}\prod_{i=1}^{n_T} X_i \cdot \exp\left(-\int_0^T \lambda(t)\mathrm{d}t\right)\right)\right] && \text{(S.18)}\\
&= n_T \log\Delta + \mathrm{E}_Z\left[\sum_{i=1}^{n_T}\log X_i\right] - \int_0^T \lambda(t)\mathrm{d}t. && \text{(S.19)}
\end{aligned}
$$

$\mathrm{E}_Z\left[\sum_{i=1}^{n_T}\log X_i\right]$ can be further calculated as follows, where we assume that event x_i is generated by the $z(i)$th event; that is, event $x_{z(i)}$.

$$
\begin{aligned}
&\mathrm{E}_Z\left[\sum_{i=1}^{n_T}\log X_i\right]\\
&= \sum_Z\left(\Pr\left[Z\right]\sum_{i=1}^{n_T}\log X_i\right)\\
&= \sum_Z\left(\prod_{i=1}^{n_T}\pi_{i,z(i)}\sum_{i=1}^{n_T}\log X_i\right)\\
&= \sum_{i=1}^{n_T}\sum_Z\left(\left(\prod_{i=1}^{n_T}\pi_{i,z(i)}\right)\log X_i\right)\\
&= \sum_{i=1}^{n_T}\left\{\sum_{Z\text{ s.t. } Z_{i,i}=1}\left(\left(\prod_{i=1}^{n_T}\pi_{i,z(i)}\right)\log X_i\right) + \sum_{Z\text{ s.t. } Z_{i,i}=0}\left(\left(\prod_{i=1}^{n_T}\pi_{i,z(i)}\right)\log X_i\right)\right\}\\
&= \sum_{i=1}^{n_T}\left\{\sum_{Z\text{ s.t.}Z_{i,i}=1}\left(\left(\pi_{i,i}\prod_{k\neq i}\pi_{k,z(k)}\right)\log\mu_i\right)\right.\\
&\qquad\left. + \sum_{j;t_j<t_i}\left(\pi_{i,j}\sum_{Z\text{ s.t.}Z_{i,j}=1}\left(\prod_{k\neq i}\pi_{k,z(k)}\right)\log\left(\eta(t_j)h(t_i-t_j)\right)\right)\right\}\\
&= \sum_{i=1}^{n_T}\left\{\pi_{i,i}\cdot 1\cdot\log\mu_i + \sum_{j;t_j<t_i}\left(\pi_{i,j}\cdot 1\cdot\log\left(\eta(t_j)h(t_i-t_j)\right)\right)\right\}\\
&= \sum_{i=1}^{n_T}\left\{\pi_{i,i}\log\mu_i + \sum_{j;t_j<t_i}\left(\pi_{i,j}\log\left(\eta(t_j)h(t_i-t_j)\right)\right)\right\}. && \text{(S.20)}
\end{aligned}
$$

S.2 Validity of our estimation

This section discusses the validity of our estimates. For the evaluation, we considered the following properties of point processes: If the intensity function of the point process $\{t_i\}$ is $\lambda(t)$, then the point process of $\{\int_0^{t_i} \lambda(t)\mathrm{d}t\}$ follows a stationary Poisson process with an intensity 1 (constant) [2,3]. Therefore, the random variables $\{\int_0^{t_i} \lambda(t)\mathrm{d}t\}$ must follow a uniform distribution over the observation period $[0, T]$ for true $\lambda(t)$.

The empirical and theoretical cumulative density functions (CDFs) of $\{\int_0^{t_i} \lambda(t)\mathrm{d}t\}$ are shown for 2, 16 and 30 March 2020, as an example, in Figs. S.1-S.6. In each figure, i/N (empirical CDF) and $\int_0^{t_i} \lambda(t)\mathrm{d}t/T$ (theoretical CDF) are plotted against $\int_0^{t_i} \lambda(t)\mathrm{d}t$, where N is the number of transactions. The theoretical CDF is on a diagonal line according to this definition. The thin lines above and below the diagonal line represent the values of $i/N + 1.62/\sqrt{N}$ and $i/N - 1.62/\sqrt{N}$, respectively. By Kolmogorov and Smirnov (KS) test, the null hypothesis that "$\int_0^{t_i} \lambda(t)\mathrm{d}t$ follows the uniform distribution" is rejected with the significance level 0.01, if the empirical CDF is plotted outside $[i/N + 1.62/\sqrt{N},\ i/N - 1.62/\sqrt{N}]$.

For almost all point processes analyzed, the null hypothesis was rejected, and we could not show that $\{\int_0^{t_i} \lambda(t)\mathrm{d}t\}$ follows the stationary Poisson process with an intensity 1 for the estimated $\lambda(t)$, whereas the empirical CDFs were relatively close to the diagonal line for many point processes analyzed. The deviation of the empirical CDFs from the diagonal line, or misestimation of $\lambda(t)$, is primarily owing to the simplification of our model. We estimated the values of the background intensity $\mu(t)$ and branching ratio $\eta(t)$ for $t = k\delta$ ($k = 0, 1, ..., 10$, $\delta = 15$ min), and obtained $\mu(t_i)$ and $\eta(t_i)$ as the occurrence of the ith transaction by linear interpolation. Therefore, in this procedure, instantaneous changes in the values of $\mu(t)$ or $\eta(t)$ are smoothened. Many empirical CDFs for the point processes in the morning and afternoon sessions were concave and convex, respectively. This tendency suggests that our estimation did not sufficiently capture the instantaneous salience in $\mu(t)$ or $\eta(t)$ at the beginning and end of the morning and afternoon sessions.

We can estimate $\mu(t)$ and $\eta(t)$ more precisely by assuming more complex functions to represent the temporal changes for each point process. However, their estimation using a simple model also enabled us to analyze many point processes for various stocks by reducing computational costs and easily comparing their results. Considering the advantages of this simple model, we assumed a simple intensity function in this study.

References

[1] Hawkes AG, Oakes D. A cluster process representation of a self-exciting process. Journal of applied probability. 1974;11(3):493-503.

[2] Filimonov V, Sornette D. Quantifying reflexivity in financial markets: Toward a prediction of flash crashes. Physical Review E. 2012;85(5):056108.

[3] Wehrli A, Sornette D. Classification of flash crashes using the Hawkes (p, q) framework. Quantitative Finance. 2022;22(2):213-40.

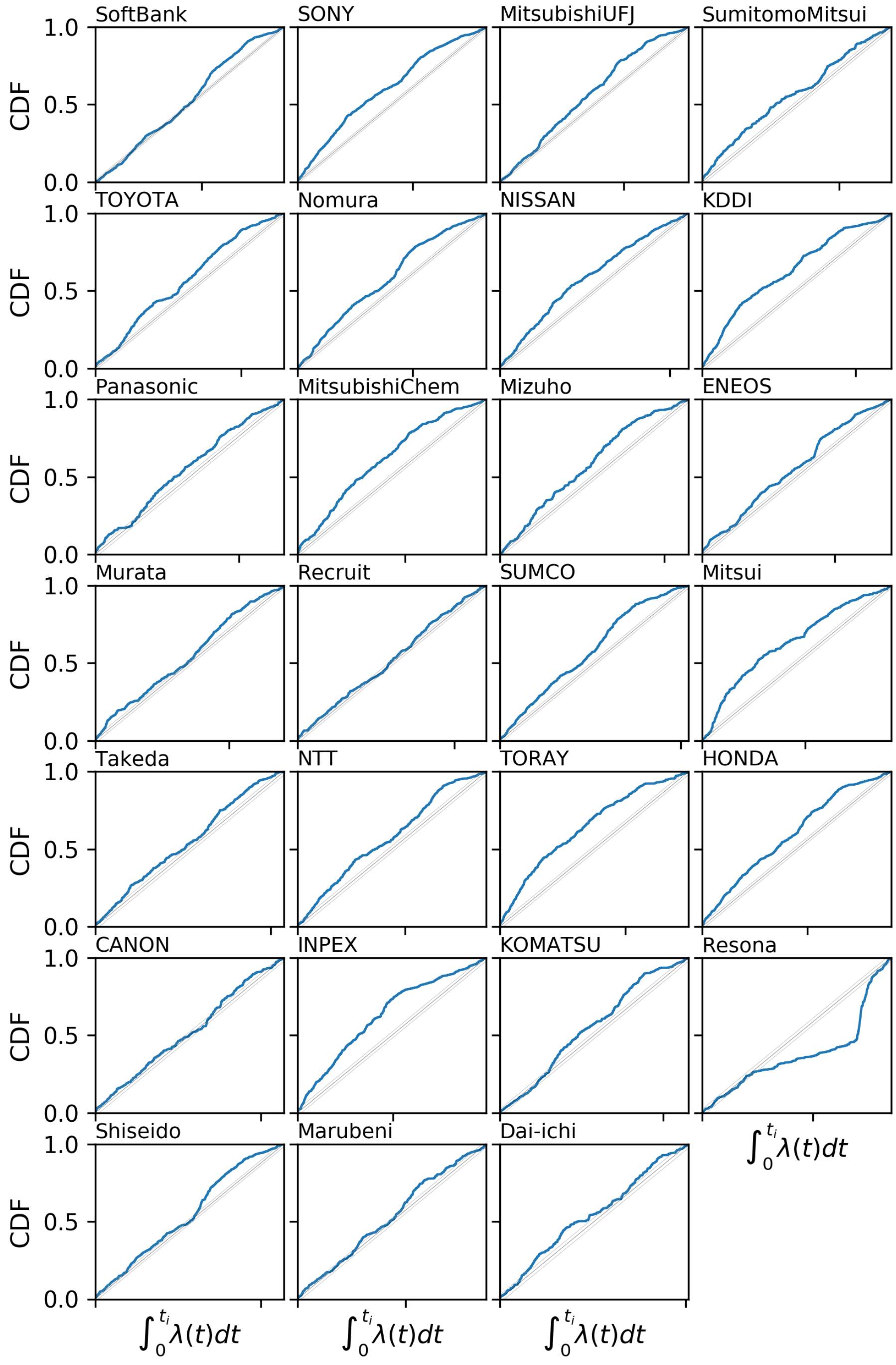

Figure S.1: CDF of $\int_0^{t_i}\lambda(t)\mathrm{d}t$ for point processes of transactions in the morning session on 2 March 2020. Each panel corresponds to the issue shown in the upper left corner of the panel. The horizontal axis shows $\int_0^{t_i}\lambda(t)\mathrm{d}t$. For the ordinate, the theoretical CDF $\int_0^{t_i}\lambda(t)\mathrm{d}t/T$ and the empirical CDF i/N are shown by the grey diagonal line and blue line, respectively.

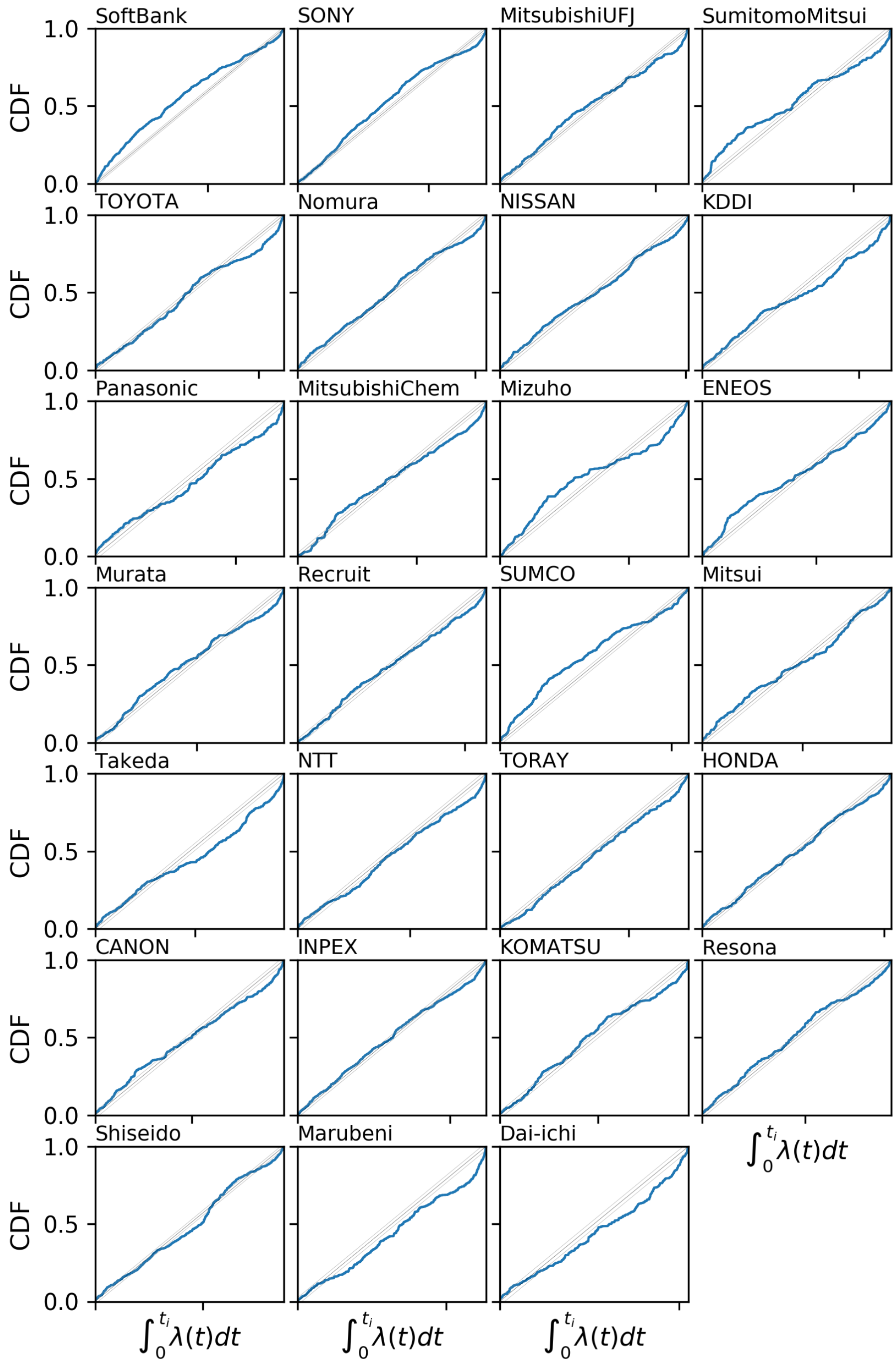

Figure S.2: CDF of $\int_0^{t_i} \lambda(t)\mathrm{d}t$ for point processes of transactions in the afternoon session on 2 March 2020. CDFs are shown in the same manner as that in Fig. S.1.

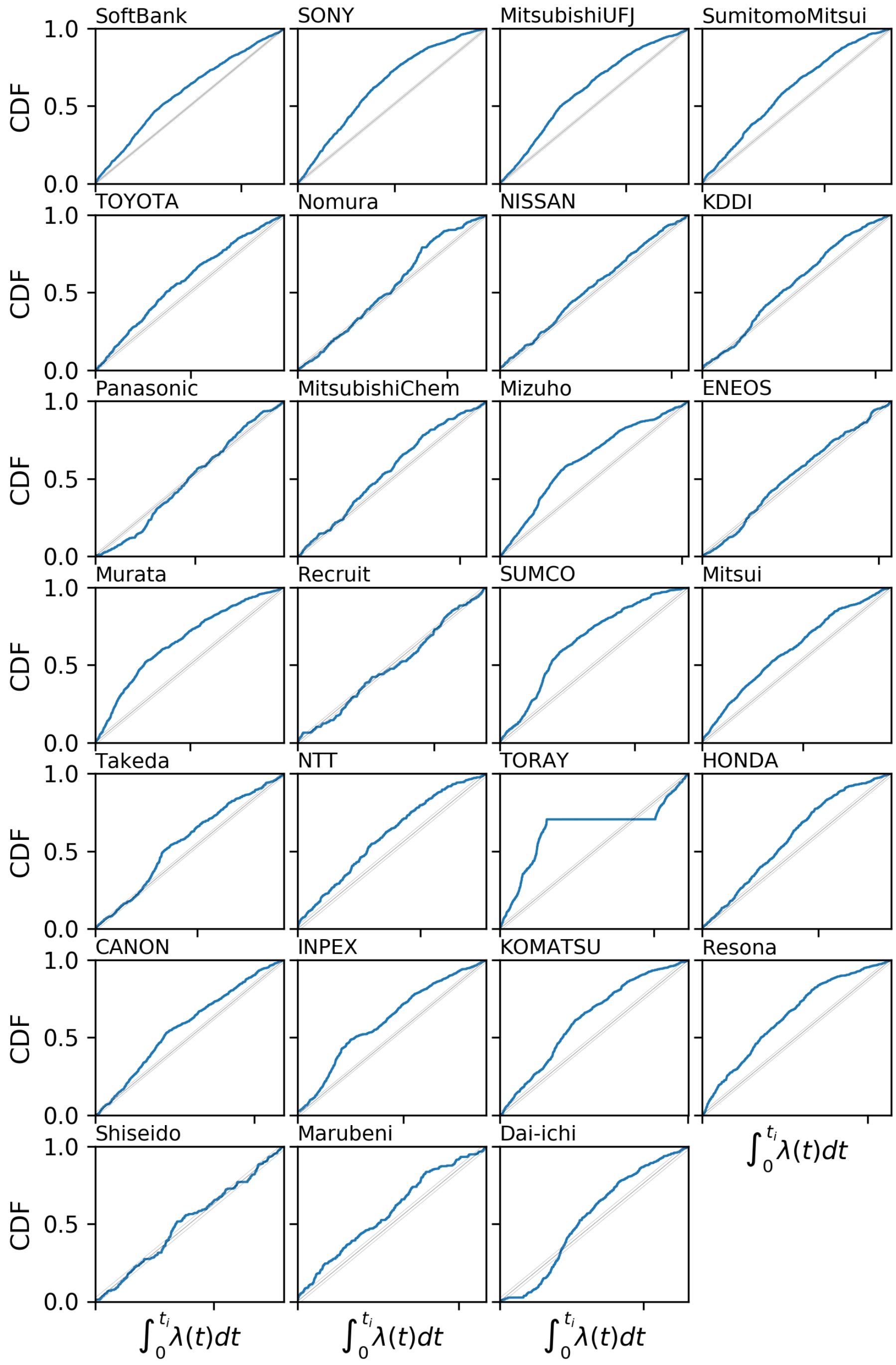

Figure S.3: CDF of $\int_0^{t_i} \lambda(t)\mathrm{d}t$ for point processes of transactions in the morning session on 16 March 2020. CDFs are shown in the same manner as that in Fig. S.1.

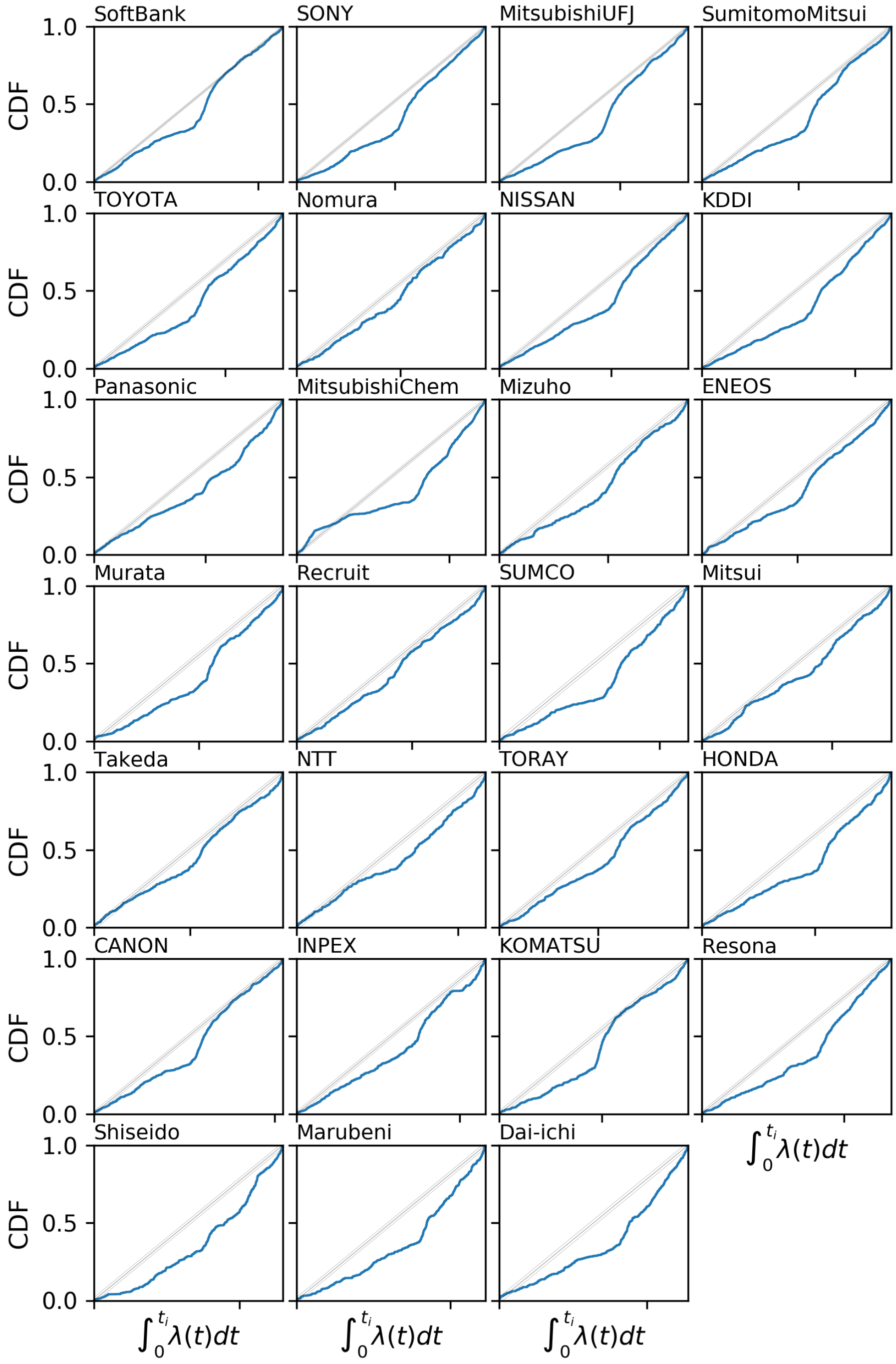

Figure S.4: CDF of $\int_0^{t_i} \lambda(t)\mathrm{d}t$ for point processes of transactions in the afternoon session on 16 March 2020. CDFs are shown in the same manner as that in Fig. S.1.

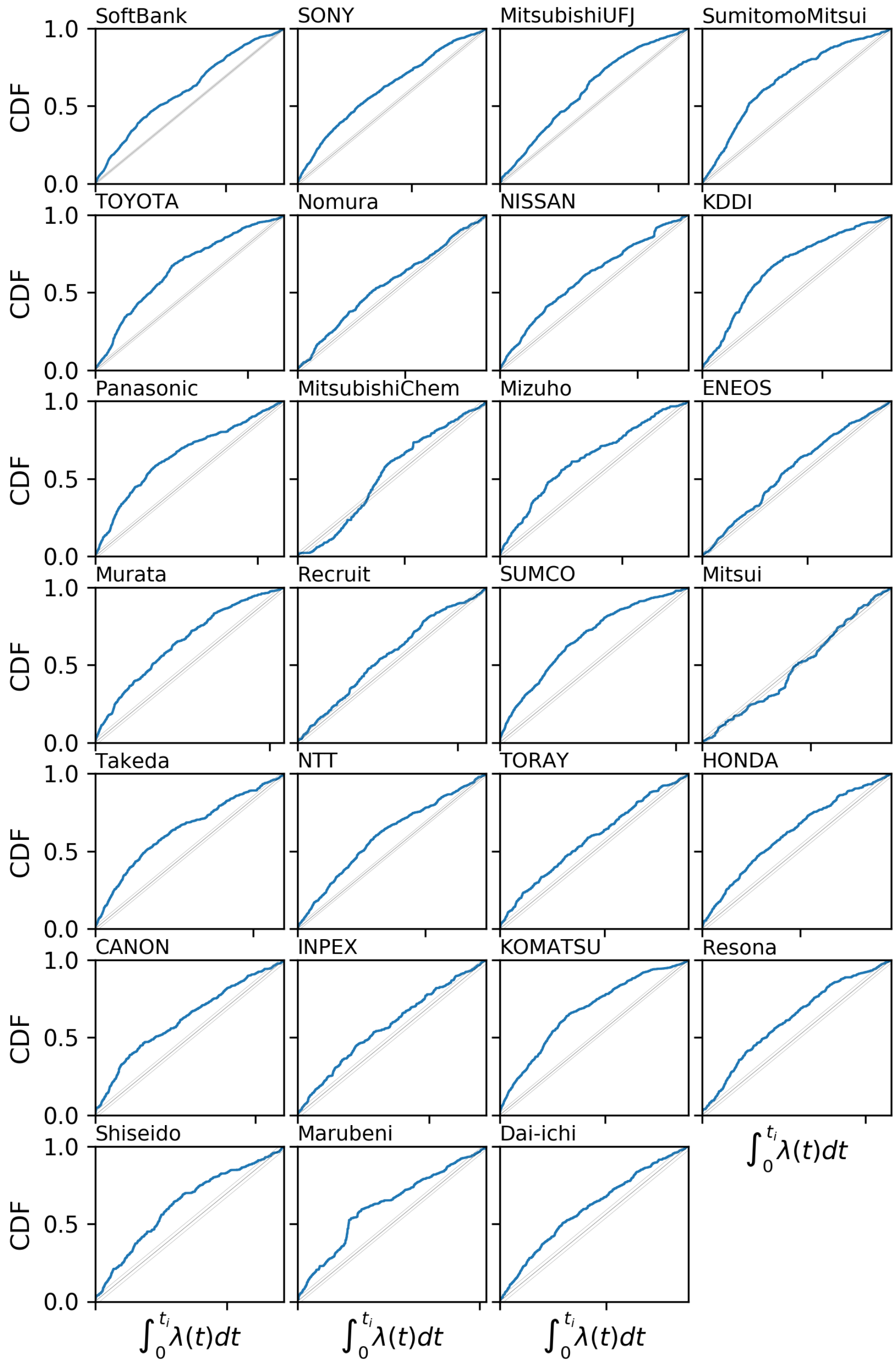

Figure S.5: CDF of $\int_0^{t_i} \lambda(t)\mathrm{d}t$ for point processes of transactions in the morning session on 30 March 2020. CDFs are shown in the same manner as that in Fig. S.1.

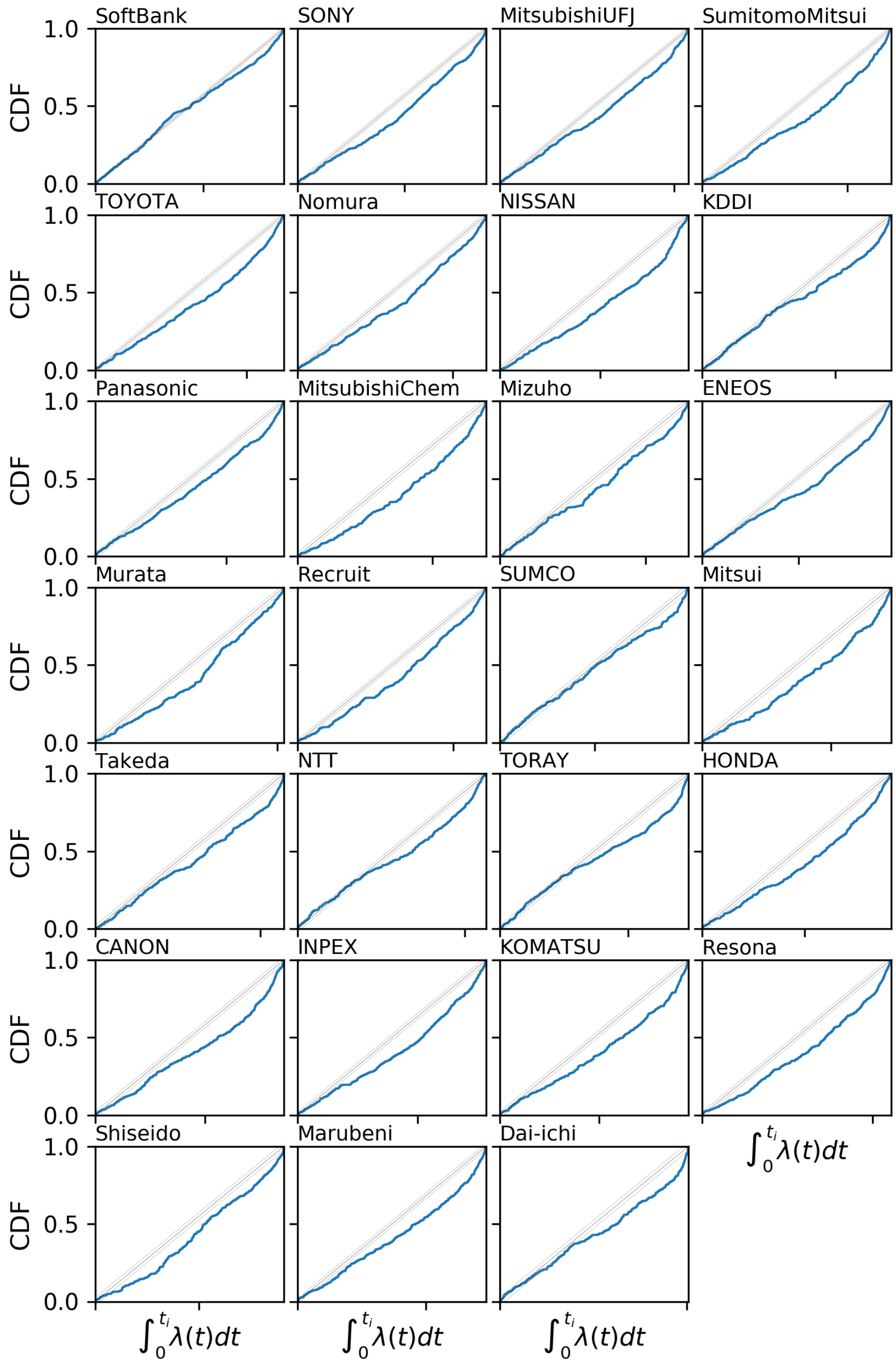

Figure S.6: CDF of $\int_0^{t_i} \lambda(t)\mathrm{d}t$ for point processes of transactions in the afternoon session on 30 March 2020. CDFs are shown in the same manner as that in Fig. S.1.
